# An RNA-Seq Screen of the *Drosophila* Antenna Identifies a Transporter Necessary for Ammonia Detection

**DOI:** 10.1371/journal.pgen.1004810

**Published:** 2014-11-20

**Authors:** Karen Menuz, Nikki K. Larter, Joori Park, John R. Carlson

**Affiliations:** 1 Department of Molecular, Cellular and Developmental Biology, Yale University, New Haven, Connecticut, United States of America; 2 Interdepartmental Neuroscience Program, Yale University, New Haven, Connecticut, United States of America; University of California Davis, United States of America

## Abstract

Many insect vectors of disease detect their hosts through olfactory cues, and thus it is of great interest to understand better how odors are encoded. However, little is known about the molecular underpinnings that support the unique function of coeloconic sensilla, an ancient and conserved class of sensilla that detect amines and acids, including components of human odor that are cues for many insect vectors. Here, we generate antennal transcriptome databases both for wild type *Drosophila* and for a mutant that lacks coeloconic sensilla. We use these resources to identify genes whose expression is highly enriched in coeloconic sensilla, including many genes not previously implicated in olfaction. Among them, we identify an ammonium transporter gene that is essential for ammonia responses in a class of coeloconic olfactory receptor neurons (ORNs), but is not required for responses to other odorants. Surprisingly, the transporter is not expressed in ORNs, but rather in neighboring auxiliary cells. Thus, our data reveal an unexpected non-cell autonomous role for a component that is essential to the olfactory response to ammonia. The defective response observed in a *Drosophila* mutant of this gene is rescued by its *Anopheles* ortholog, and orthologs are found in virtually all insect species examined, suggesting that its role is conserved. Taken together, our results provide a quantitative analysis of gene expression in the primary olfactory organ of *Drosophila*, identify molecular components of an ancient class of olfactory sensilla, and reveal that auxiliary cells, and not simply ORNs, play an essential role in the coding of an odor that is a critical host cue for many insect vectors of human disease.

## Author Summary

Olfaction underlies the attraction of insect pests and vectors of disease to their plant and human hosts. In the genetic model insect *Drosophila*, the neuronal basis of odor coding has been extensively analyzed in the antenna, its major olfactory organ, but the molecular basis of odor coding has not. Additionally, there has been little analysis of any olfactory cells other than neurons. We have undertaken a comprehensive and quantitative analysis of gene expression in the *Drosophila* antenna. This analysis revealed a surprisingly broad dynamic range of odor receptor and odor binding protein expression, and unexpected expression of taste receptor genes. Further analysis identified 250 genes that are expressed at reduced levels in a mutant lacking an evolutionarily ancient class of sensilla, antennal hairs housing neurons that respond to human odors. One of these genes, a transporter, is expressed in non-neuronal cells but is essential to the response of a neuron to ammonia, a key cue for insect vectors of disease. A mutation in this transporter can be rescued by its mosquito homolog. While many studies of sensory coding consider the neural circuit in isolation, our analysis reveals an essential role for an auxiliary cell.

## Introduction

Olfaction is a critical sensory modality for insects, as it acts in the identification of food and mates. Insect olfaction is of global significance in that many insects that transmit disease, and many agricultural pests that ravage the world's food supply, find their human or plant hosts through olfactory cues [1], [2].

**Figure 1:**
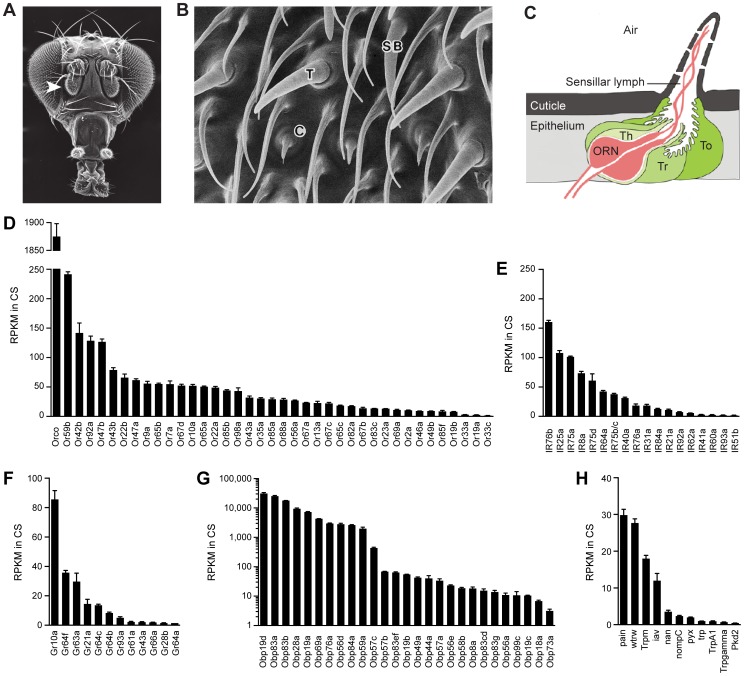
Chemosensory gene expression in the wild-type antennal third segment. (A) Third antennal segment (arrowhead) on a *Drosophila* head. (B) Scanning electron micrograph of the antennal surface with a coeloconic sensillum (C), trichoid sensillum (T) and small basiconic sensillum (SB) labeled. (C) Diagram of a generic sensillum containing an olfactory receptor neuron (ORN) whose dendrite is surrounded by sensillar lymph. The sensillum and antennal surface is covered with a cuticle, and pores in the sensillum cuticle permit airborne odors to enter the sensillum lymph. The sensillum is separated from other sensilla by the epithelium. Auxiliary cells, including tormogen (To), trichogen (Tr) and thecogen (Th) cells, surround the ORN. (D) Members of the *Or* and (E) *IR* olfactory receptor gene families detected in the Canton-S (CS) third antennal segment with at least 1 read per million mapped reads (RPM) in each of three samples. Genes are listed by decreasing reads per million mapped reads per kilobase of gene length (RPKM). Two *IR* genes, *IR75b* and *IR75c,* were annotated as a single gene, *CG14586,* at the time of the gene mapping (Dataset S1), and are therefore represented by a single bar in the graph. (F) *Gr* genes. (G) *Obp* genes. (H) *Trp* family genes. (A) is from http://cedar.bio.indiana.edu/~ggrumbli/highrespackage, (B) is from [3], and (C) is adapted from [116].

The most extensively studied insect olfactory system is that of *Drosophila*. The third antennal segment of the fly is covered with three main morphological classes of olfactory sensilla: basiconic, trichoid, and coeloconic sensilla (Figure 1A, B) [3], [4]. Each sensillum contains the dendrites of up to four olfactory receptor neurons (ORNs) with different odorant response profiles. ORNs of basiconic sensilla respond to food odors, including many esters and alcohols; ORNs of trichoid sensilla respond to fly odors; ORNs of coeloconic sensilla respond to many amines and carboxylic acids [5]–[8]. Additional olfactory sensilla are located in the sacculus, a three-chambered pit under the antennal surface, and on the maxillary palp, an organ that extends from the proboscis [4].

Odor receptors of the Or family underlie the responses of basiconic and trichoid sensilla [7], [9]. Most individual ORNs in these sensilla express one of ∼60 Ors as well as Orco, a related co-receptor [10]–[12]. ORNs of coeloconic sensilla express members of the IR family of ionotropic receptors, an evolutionarily ancient class of ∼60 receptors, of which some are co-receptors [13]–[15].

The *Drosophila* olfactory organs have been the subjects of detailed systematic anatomical and physiological studies, but their molecular biology has not been extensively characterized. For example, remarkably little is known about how the molecular underpinnings of olfaction differ among different classes of sensilla, other than that they express different receptors. The molecular basis of olfaction in coeloconic sensilla is of particular interest in that these sensilla – and their morphological counterparts in mosquitoes - respond to many human volatiles, including carboxylic acids and ammonia [5], [6], [16]–[18]. Molecular targets specific to sensilla could be particularly useful in designing novel approaches to the control of insect vectors and the diseases they carry.

Although ORNs have received a great deal of attention, the non-neuronal cells in olfactory sensilla are largely unexplored. Olfactory sensilla contain three or four auxiliary cells (Figure 1C) [19]. These cells express odorant binding proteins (OBPs) and odorant-degrading enzymes (ODEs), which bind and degrade odorants, respectively [20]–[22]. However, the contributions of these cells to odor coding *in vivo* are poorly understood.

Here we have addressed these major gaps in our knowledge. We began by carrying out a molecular screen for genes whose expression is highly enriched in coeloconic sensilla, including genes of both neurons and auxiliary cells. Specifically, we carried out an RNA-Seq analysis of antennae from both wild type flies and *ato* mutants, which lack coeloconic sensilla but which retain basiconic and trichoid sensilla [23]. The screen identified 250 genes severely depleted in *atonal* antennae. The proteins encoded by these genes are highly enriched in receptors, ion channels, and transporters. One of these genes encodes a member of a highly conserved ammonium transporter family found in bacteria, yeast, and plants. We have named the gene *Amt (Ammonium transporter)* and have found that on the antennal surface it is exclusively expressed in auxiliary cells of one kind of coeloconic sensillum. Genetic and electrophysiological analysis revealed that despite its non-neuronal expression, *Amt* is required for the ammonia response of an olfactory neuron in this sensillum. These results illustrate *in vivo* an essential contribution of a non-neuronal auxiliary cell to an olfactory circuit.

## Results

### The olfactory receptor repertoire of the antenna

We profiled the transcriptome of the third segment of the *Drosophila* antenna by mRNA sequencing (RNA-Seq). Dissected third segments from Canton-S (CS) flies were divided into three biological replicates and analyzed independently. Of ∼24 million reads, 85% could be aligned to the *Drosophila* genome, and 90% of the aligned reads mapped to unique genes in the FlyBase annotation set (Table S1 and Dataset S1). Of 14,078 genes in the FlyBase set, 8,646 (61%) were detected at ≥1 read per million mapped reads (RPM) in all three samples, and we designated these as the set of third antennal segment genes (Dataset S1).

We found expression of 39 *Or* genes (Figures 1D and S1). These results are in good agreement with earlier *in situ* hybridization, *GAL4,* and RT-PCR analysis of *Or* expression in the antenna [10], [11], [24], [25]. We did not observe expression of *Or33b* or *Or49a*, which had been detected by *in situ* hybridization in some but not all previous studies [10], [11], [24], [25]. Expression of these genes may be sensitive to the genetic background. Transcripts of two of the detected *Or* genes, *Or46a* and *Or33c*, had not previously been identified in the antenna by *in situ* hybridization, although they had been found by RT-PCR [10], [11], [24], [25].

We detected expression of 19 *IR* genes, 17 of which have previously been observed in the antenna (Figures 1E and S1) [5], [14], [15]. Our analysis confirmed expression of *IR60a*, which had been identified by RT-PCR but not by *in situ* hybridization analysis [14]. We also identified reads representing *IR51b* and *IR62a*, which have not previously been found in the antenna. Both of these genes are expressed at low levels, and reside in introns of more highly-expressed antennal genes; these reads could derive from unspliced transcripts of the surrounding genes rather than independent *IR* transcripts.

We considered the purity of our antennal preparations. There was little if any contamination with maxillary palps: most *Or* genes expressed in the maxillary palp [10], [11], [26] were not detected in our analysis of the antenna. Moreover, there was little contamination with second antennal segments: many highly abundant transcripts in the second segment were not found in our third segment dataset (Figure S2) [27].

### Large dynamic range of olfactory receptor expression

The dynamic range of *Or* expression is striking (Figure 1D). *Orco*, which encodes the co-receptor of canonical Ors, was detected at a level 43-fold higher than the mean level of the 38 detected canonical *Or* genes: 1875 reads per kilobase per million mapped reads (RPKM) vs. 44 RPKM, respectively. This expression ratio supports a 1∶1 stoichiometry of Orco and canonical Ors. Among the canonical *Or* genes, expression ranged from 242 RPKM in the case of *Or59b* down to 1.4 RPKM in the case of *Or33c*. Thus the levels of canonical *Ors* varied over a 170-fold range.

Does the *Or* expression level correlate with sensillum or neuron type? *Ors* of large basiconic sensilla are expressed at higher levels (94±26 RPKM; n = 8 genes, SEM) than those of small basiconic sensilla (30±6 RPKM; n = 15 genes) or trichoid sensilla (34±10 RPKM; n = 12 genes) (p<0.005 and p<0.05, respectively, ANOVA) (Figure S3). We note with interest that in eight of nine basiconic sensillum types in which the receptor-to-neuron map is established [9]–[11], [28]–[30], the *Or* that is expressed at the highest level maps to the A neuron, which yields the action potential with the greatest amplitude (Figure S3).

*IR* genes also showed a wide dynamic range of expression (Figure 1E). The *IRs* believed to function as co-receptors (*IR76b, IR25a,* and *IR8a*) [13] are expressed at levels that are substantially higher than the mean level of the other *IRs*. Among the other *IRs*, the highest expression level was 101 RPKM (*IR75a*), and several were expressed at less than 3 RPKM.

### Unexpected gustatory receptor expression in the antenna

We were surprised to detect expression of 12–14 *Gustatory Receptor (Gr)* genes in the antenna (Figures 1F and S4). Many of these Grs are sugar receptors, such as Gr43a, implicated in fructose detection, and Gr61a, implicated in the detection of multiple sugars [31]–[33]. These antennal Gr genes also include *Gr64f, Gr64c, Gr64b, Gr64a*, all of which lie in a cluster of genes and all of which have been implicated in sugar reception [32], [34]–[37]. Two additional genes of the cluster, *Gr64e and Gr64d*, were detected at ∼2 RPKM, but quantitative analysis of their expression is made difficult by complications in their annotation and technically they do not meet our formal criteria for antennal expression. *Gr64f* is expressed at 36 RPKM, a level higher than 22 of the antennal *Or* genes. We verified the antennal expression of the 12 Grs expressed in the RNA-Seq dataset, as well as *Gr64e* and *Gr64d*, using qRT-PCR on additional sets of CS antennal cDNA (Figure S5A). The relative expression levels of these *Grs* as measured by qRT-PCR were similar to those observed by RNA-Seq (Figures 1F and S5A).

The *Gr* gene that is most abundantly expressed in the antenna is *Gr10a* (86 RPKM), which is of unknown function and has been previously mapped to the ab1D olfactory neuron [9], [10]. Also detected are *Gr63a* and *Gr21a*, which are believed to form a heteromeric receptor that mediates response to CO_2_ and certain odorants in ab1C neurons [38]–[40], *Gr28b*, which has recently been shown to act as a thermosensor in the antenna [41], and *Gr66a* and *Gr93a*, both implicated in the detection of bitter compounds in the taste system [42]–[44].

### *OBP* and *Trp* expression in the antenna

The two most highly expressed antennal genes are *OBPs (Odorant Binding Proteins)*. Moreover, five of the 10 most highly expressed genes of the third antennal segment are *OBPs* (Figure S6). OBPs are small, secreted proteins of 13–28 kDa that are highly divergent in sequence and are found in the fluid that bathes the dendrites of chemosensory neurons. Their function is unclear, but some have been found to bind odorants and may carry them to receptors in the dendrites of ORNs [20]–[22]. One OBP has been shown to contribute to ORN signaling in *Drosophila* [45].

RNA-Seq revealed antennal expression of 27 of the 52 members of the OBP family. Their expressions levels varied over a remarkable range, spanning four orders of magnitude (Figures 1G (note the logarithmic scale) and S4). Most OBPs fall into two groups: 10 are expressed at levels exceeding 1,000 RPKM, while 16 are at levels less than 70 RPKM. Abundance may correlate with tissue-specificity of expression: most of the *OBPs* expressed at lower levels have also been found to be expressed in taste tissue, while most of the abundantly expressed *OBPs* have not been detected outside the antenna [46]–[49].

We detected expression of 11 of 16 members of the *Transient receptor potential (Trp)* family of ion channel genes (Figures 1H and S4). Trp channels have been implicated in sensory functions, including chemosensation, in both mammals and insects [50]–[53]. Some Trp channels in *Drosophila* have been localized to small subsets of antennal neurons [52]–[56], consistent with the low levels of expression we observe in RNA-Seq.

### A screen for genes highly enriched in coeloconic sensilla reveals 250 candidates

**Figure 2:**
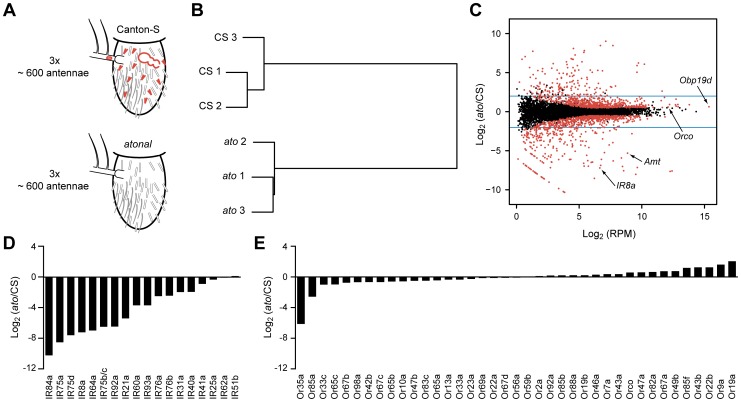
Transcriptome profiling identifies genes differentially expressed in CS and *ato* third antennal segments. (A) Schematic of the RNA-Seq screen in which three independent biological samples of third antennal segments from both CS and *ato* flies were collected. *ato* flies selectively lack coeloconic sensilla (small red hairs), aristal neurons (red oval in the arista, which extends to the left), and the multi-chambered sacculus (red bubble-like structure), which contains coeloconic sensilla. (B) Hierarchical cluster analysis of each sample based on their gene expression, showing that the expression patterns cluster by genotype. (C) Each of the antennal-expressed genes (dots) is plotted based on its average expression level in RPM and the log_2_ of the expression ratio between *ato* and CS flies. Most genes cluster around 0, indicating no change in expression. Red dots indicate differentially expressed genes (FDR <0.01), and the blue horizontal lines indicate a four-fold change level. Several genes of interest are indicated. The diagonal “line” of red dots in the bottom left consists of genes with no expression in *ato* (see Materials and Methods). (D) Nearly all of the *IR* genes are greatly reduced in *ato* antennae, consistent with their known localization to coeloconic sensilla. (E) In contrast, most of the *Or* genes expressed in CS antennae show little if any reduction in *ato.* We note that the fold-change values are approximate and are especially difficult to assess for genes that are expressed at extremely low levels in *ato*. (Figure S1). See Methods for further details.

We carried out a screen for genes specifically expressed in coeloconic sensilla. Genes expressed in coeloconic sensilla, but not in other olfactory sensilla or elsewhere in the antenna, seem more likely to contribute directly to olfactory signaling than to represent general housekeeping functions. Accordingly, we performed an RNA-Seq analysis of the third antennal segments of flies lacking Atonal (Ato), a transcription factor that specifies coeloconic sensilla (Table S1) [23]. *ato* antennae lack coeloconic sensilla on the antennal surface, while maintaining normal numbers of basiconic and trichoid sensilla (Figure 2A) [23]. *ato* mutants also lack the sacculus, a three-chambered sensory cavity that houses additional coeloconic sensilla as well as a few sensilla that may have hygroreceptor or thermoreceptor function [23]. A small number of neurons in the arista, a feather-like appendage of the antenna, and a number of glial cells are also missing [23], [57].

The transcriptional profiles of third antennal segments of *ato* and wild type were distinct: the profiles of three independent *ato* samples were much more similar to each other than to the three samples of wild type (Figure 2B). However, 95% of genes detected in the CS wild type antennae were also found in all three samples of *ato* antennae (Dataset S1). Moreover, most antennal genes are expressed at comparable levels in the two genotypes, as revealed by calculating the ratio of expression in the two genotypes, and plotting this ratio against the expression level of each antennal gene (Figure 2C). The great majority of genes clustered around a ratio of 1.

As a positive control to test the efficacy of the screen, we examined the expression of *IRs*, which are expressed in coeloconic sensilla. Of the 17 previously identified antennal *IRs*, levels of 15 were reduced in *ato* antennae by a factor greater than ∼4, and some were not detected in *ato* antennae (Figures 2D and S1). These results validate the efficacy of the screen. We note, however, a surprising exception to the general pattern of reduction in *IR* gene expression: *IR25a*, which encodes an IR co-receptor, showed only a small reduction, to a level ∼1/3^rd^ lower than in wild type. We confirmed that substantial *IR25a* expression remains in *ato*, using RT-PCR with three additional independent sets of CS and *atonal* antennae, whereas expression of a more typical *IR* gene, *IR8a*, is lost (Figure S5B). These results suggest that *IR25a* is expressed in either basiconic or trichoid sensilla, in addition to coeloconic sensilla. Likewise, low expression levels of *IR76b* and a few other *IRs* in *ato* mutants (Figure S1) suggest some degree of expression in non-coeloconic sensilla, in addition to their expression in coeloconic sensilla. We note that *IR60a* expression, which had not been found previously by *in situ* hybridization, was reduced by more than 10-fold in *ato*, consistent with its identification as a *bona fide* antennal *IR* in wild type. By contrast, levels of *IR51b* and *IR62a,* which reside in introns, were nearly unchanged.

As a negative control to test the reliability of the screen, we examined the expression of *Or* genes, nearly all of which are expressed in basiconic and trichoid sensilla, but not coeloconic sensilla. In *ato* mutants there was little if any reduction in expression of any of the 39 *Or* genes, with two exceptions (Figures 2E and S1). *Or35a* levels were reduced by 70-fold, which is fully consistent with the loss of coeloconic sensilla, since *Or35a* is the one *Or* gene that is expressed in coeloconic sensilla [6], [11]. Levels of *Or85a* also declined, which may be related to its location in the genomic region removed by the *ato* deficiency, *Df(3R)p13* (see Materials and Methods) [58].

**Figure 3:**
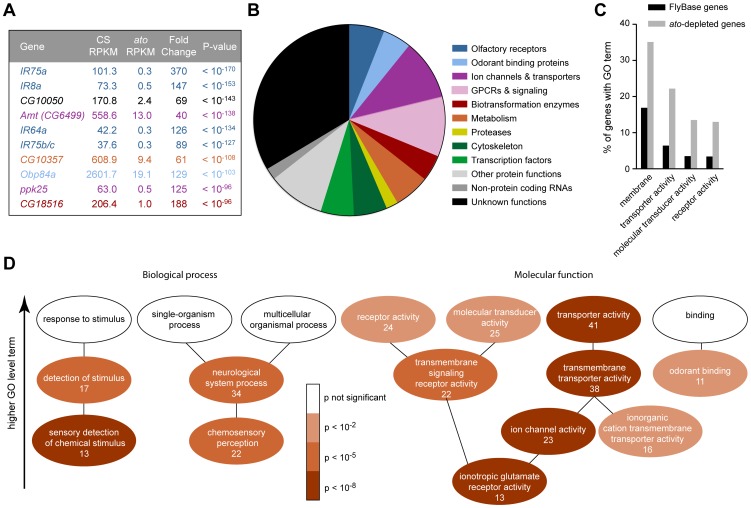
Functional categorization of 250 genes depleted in *ato.* (A) The ten genes that show the most signification depletion in *ato*, based on p-value. Each is color-coded according to the categories in panel (B). (B) Categorization of the 250 genes that show at least four-fold reduction in *ato* with a FDR <0.01. Putative functions were determined by examination of automated gene annotation and BLAST searches to identify similar proteins. (C) Gene Ontology (GO) term analysis using the program AmiGO identified four level two terms that were significantly enriched among the 250 *ato*-depleted genes compared to all *D. melanogaster* genes listed in FlyBase. See Figure S7 for the complete analysis of all level two GO terms. (D) Significance of enrichment of lower level terms, which form a subset of higher level terms (*e.g.* “detection of stimulus” is a subset of “response to stimulus”). Lower level terms were selected to illustrate the types of functions most enriched in the dataset. The number of *ato*-depleted genes annotated with each GO term is indicated. We note that the ion channel genes depleted in *ato* include two members of the *ppk* family, *ppk25* and *ppk10*, two members of the *Trp* family, *nanchung* and *inactive*, and four potassium channel genes: *shaw-like (shawl), TWIK-related acid-sensitive K^+^ channel (Task7), Inwardly rectifying potassium channel 1 (Irk1)*, and *CG1756*. Depleted genes described by “transmembrane signaling receptor activity” included six GPCRs: *5-HT2, 5-HT7, CG43795, frizzled 3 (fz3), Pigment-dispersing factor receptor (Pdfr),* and *CG18208*.

Having thus validated the screen, we carried out a statistical analysis to identify the set of genes that are differentially expressed in the two genotypes. We identified 1,490 differentially expressed genes (red dots in Figure 2C, Dataset S1) when the false discovery rate (FDR) was set at 1%. Levels of 803 of these genes were reduced in *ato*, as reflected by their negative log_2_(*ato*/CS) values. The 10 genes that were reduced with the greatest statistical significance include four *IRs*, *Obp84a*, and *ppk25*, all of which are specifically expressed in coeloconic sensilla (Figure 3A)[5], [15], [59], [60]. Given that levels of nearly all of the 17 known antennal *IRs* are reduced at least 4-fold, we analyzed further the subset of 250 genes that were at least 4-fold reduced in *ato* antennae (Dataset S1).

### Many of the enriched genes are associated with transduction and transport

Most of the 250 genes that were at least 4-fold reduced in *ato* antennae have not been well characterized in *Drosophila*. We carried out BLAST searches with each of them to gain information with which to perform an initial classification. Functions could be predicted for two-thirds of the genes. Functional categories included olfactory receptors, ion channels, transporters and transcription factors, among others; the categories are not necessarily mutually exclusive and were chosen to illustrate the functional diversity of the 250 genes (Figure 3B). One-third of the genes could not be classified with confidence. They had no known function and had little or no similarity to genes with known function in other organisms.

Are the genes identified by the screen enriched for genes of any functional classes, or do they represent a random sample of all possible classes? To address this issue quantitatively, we examined their annotations with Gene Ontology (GO) terms. GO terms refer to a cellular component, biological process, or molecular function with which a protein is associated. Moreover, GO terms are arranged in a hierarchy, in which more specific terms occupy lower levels of the hierarchy. Of 250 genes, 185 had previously been assigned GO terms. When this subset of genes was compared to all FlyBase genes with GO annotations, we found that some high level GO terms were significantly enriched, for example the cellular component “membrane” (p<10^−5^), and the molecular functions “transporter activity” (p<10^−8^), “molecular transducer activity” (p<10^−4^) and “receptor activity” (p<10^−4^) (Figures 3C and S7), as detailed below.

Among terms describing biological processes, there was enrichment for genes annotated for roles in the “sensory detection of chemical stimulus” (7.0% of *ato*-depleted genes vs. 0.5% of FlyBase genes, p<10^−8^), “chemosensory perception” (11.9% of *ato*-depleted genes vs. 1.9% of FlyBase genes, p<10^−7^), and “neurological system process” (18.4% of *ato*-depleted genes vs. 5.9% of FlyBase genes, p<10^−5^)(Figure 3D). This enrichment is consistent with the loss of olfactory structures in *ato* antennae.

With respect to molecular function, there was particularly strong enrichment for genes described by the term “ionotropic glutamate receptor activity” (7.0% of *ato*-depleted genes vs. 0.3% of FlyBase genes, p<10^−11^), which is consistent with the loss of *IRs* in *ato* antennae (Figures 2D and 3D). These *IR* genes were a subset of the 22 genes described by “transmembrane signaling receptor activity” (11.9% of *ato*-depleted genes vs. 2.6% of FlyBase genes, p<10^−5^), which also included six GPCRs. Additionally, there was strong enrichment for genes described by the term “ion channel activity” (12.4% of *ato*-depleted genes vs. 1.7% of FlyBase genes, p<10^−10^). In addition to the *IRs*, this term included 10 ion channels that were expressed at reduced levels in *ato*, including two members of the *pickpocket (ppk)* family, two members of the *Trp* family, and four potassium channels. The ion channels were in turn a subset of 38 genes described by “transmembrane transporter activity” (20.5% of *ato*-depleted genes vs. 5.6% of FlyBase genes, p<10^−8^). This subset also contained twelve members of the solute carrier (SLC) family of transporters. One transporter is considered in detail below.

We also examined the genes that were expressed at higher, rather than lower, levels in *ato* than wild type, with an interest in understanding what other changes in antennal gene expression occur in the absence of coeloconic sensilla. Among 155 genes expressed at levels that were at least 4-fold higher in *ato* flies, 130 had been assigned GO terms. Many of these genes are associated with the immune system and defense responses, such as extracellular antimicrobial peptides (Dataset S1 and Figure S7). The increased expression of these genes may reflect a general debilitation of *ato* mutants, which exhibit a variety of developmental defects and which could conceivably suffer an increased susceptibility to microbial infections [58], [61].

### *Amt* is expressed at extremely high levels in auxiliary cells in a subset of coeloconic sensilla

One gene identified in the screen offered a particularly interesting opportunity to test whether an individual gene whose expression is depleted in *ato* in fact functions in coeloconic sensilla. This gene, *CG6499*, is expressed at extremely high levels in the wild type antenna and is severely depleted in *ato* (559 RPKM vs. 13 RPKM, Figure 3A). Its function in *Drosophila* is unexplored, but an additional virtue of this gene is that its sequence identifies it as an ammonium transporter. It encodes a predicted protein of 562 amino acids, of which an internal region of ∼410 amino acids exhibits ∼30% identity to well-characterized ammonium transporters from bacteria, yeast, and plants [62]–[64]. Ammonia is a critical olfactory cue for many insects, and is an attractive component of human odor for certain insect vectors of human disease, including mosquitoes [65]–[68]. Ammonia is primarily detected by coeloconic sensilla in *Drosophila* [5], [6], [69], and morphologically similar sensilla respond to ammonia and amines in other insect species [16]–[18], [65], [70], [71]. Based on the high sequence similarity of CG6499 to other members of the ammonium transporter family, we renamed it *Ammonium transporter (Amt).*

**Figure 4:**
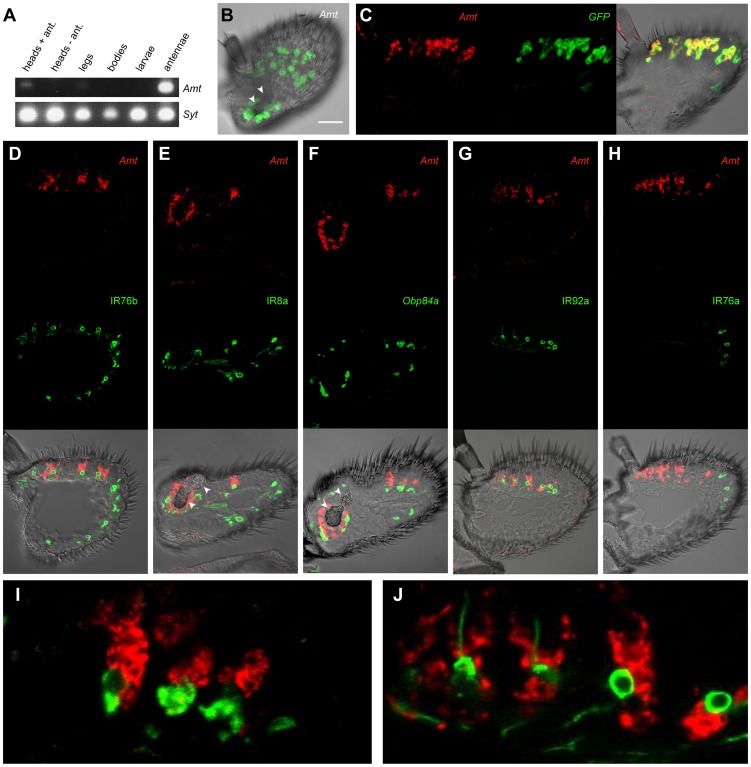
Expression of *Amt.* (A) RT-PCR analysis of *Amt* expression in CS. *Synaptogmin* was used as a positive control. (B) Whole-mount confocal image of a third antennal segment of an *Amt-GAL4; UAS-mCD8::GFP* fly. GFP expression is seen in large, amorphous auxiliary cells, but not in neurons. White arrowheads indicate the sacculus here and in panels E and F. Scale bar  = 30 µm. (C) Confocal image of an *in situ* hybridization to an antennal section from an *Amt-GAL4; UAS-mCD8::GFP* fly using antisense probes for *Amt* (red) and *GFP* (green). The two probes co-localize. (D, E, G, H), confocal images of antennal sections labeled with an antisense probe for *Amt* (red) and an antibody against GFP (green) driven by (D) *IR76b-Gal4*, (E) *IR8a-Gal4*, (G) *IR92a-Gal4*, and (H) *IR76a-Gal4*. *IR76b-Gal4* and *IR8a-Gal4* are co-receptors that label at least one ORN in each surface coeloconic sensillum type (ac1–4). *IR8a-Gal4* also labels coeloconic ORNs in the third chamber of the sacculus. *Amt* is detected in larger neighboring auxiliary cells in a subset of the coeloconic sensilla. (F) Confocal image of an *in situ* hybridization to an antennal section from a CS fly using antisense probes for *Amt* (red) and *Obp84a* (green). *Amt* is expressed in different auxiliary cells from those that express *Obp84a*, which is also expressed in coeloconic sensilla. (G) Expression of *Amt* on the antennal surface is found surrounding the IR92a ammonia receptor-expressing neurons, which are in ac1. (H) *Amt* is not detected in ac4 sensilla, which contain ORNs that express *IR76a*. (I, J) Higher magnification images of (F) and (G) respectively.

We examined the localization of *Amt* expression. As an initial step we carried out RT-PCR analysis of heads with and without antennae, legs, bodies depleted of heads and appendages, and third-instar larvae (Figure 4A). This analysis confirmed the strong expression in the antennae and revealed evidence for weak expression in legs.

Next we examined *Amt* expression in the antenna. We generated an *Amt-GAL4* line and found that it drove expression of a *UAS-mCD8::GFP* reporter in a subset of sensilla on the antennal surface and in a subset of sensilla in the sacculus (Figure 4B). Expression was localized to auxiliary cells of sensilla, rather than neurons, as determined by the morphology and large size of the labeled cells and the absence of labeled dendrites or axons. The fidelity of the *Amt-GAL4* driver was tested in double-label *in situ* hybridization experiments with probes against *GFP* and *Amt*. The two probes showed a high degree of co-localization (Figure 4C).

We then tested whether the sensilla that express *Amt* are in fact coeloconic sensilla. Specifically, we generated *GAL4* drivers for two *IR* co-receptors: *IR76b*, which is expressed in each of the four coeloconic sensilla subtypes of the antennal surface, and *IR8b*, which is expressed in all four subtypes and in coeloconic sensilla of the third chamber of the sacculus [15]. We carried out double-label experiments with a probe for *Amt* and an antibody against GFP, which was driven by the *IR-GAL4* constructs (Figure 4D, E). Double-label experiments indicated that *Amt* is expressed in a subset of coeloconic sensilla on the antennal surface and in the sacculus (Figure 4D, E). These results agree with our finding that *Amt* is depleted in *ato* antennae. The *Amt* probe and GFP antibody did not label the same cells within sensilla, however. This result is consistent with our finding that *Amt* expression is non-neuronal, because most coeloconic neurons express either *IR76b* or *IR8b*.

Given that *Amt* is expressed in auxiliary cells of sensilla, we wondered whether it is expressed in the same auxiliary cells that synthesize and secrete OBPs. Our screen had identified *Obp84a* as one of the most highly enriched genes in coeloconic sensilla (Figure 3A). We carried out a double-label experiment and found that *Obp84a* and *Amt* are expressed in different auxiliary cells (Figure 4F, I).

Finally, we wanted to determine which subset of coeloconic sensilla expresses *Amt*. Previous electrophysiological analysis had indicated that only the ac1 class of coeloconic sensilla yields large responses to ammonia [5], [6]. *IR92a* is expressed in ac1 sensilla and responds to ammonia, whereas *IR76a* is expressed in ac4 sensilla [5], [15], [69]. We found that *Amt* is expressed in the same sensilla as *IR92a-GAL4,* and not in the same sensilla as *IR76a-GAL4* (Figure 4G, H). Furthermore, neurons labeled by *IR92a-GAL4* were consistently in close proximity to *Amt*-labeled auxiliary cells (Figure 4G, J). In summary, we conclude that *Amt* is specifically expressed in all or most ac1 sensilla, in good agreement with previous physiological results.

### *Amt* plays a critical role in ammonia response of ac1 sensilla

**Figure 5:**
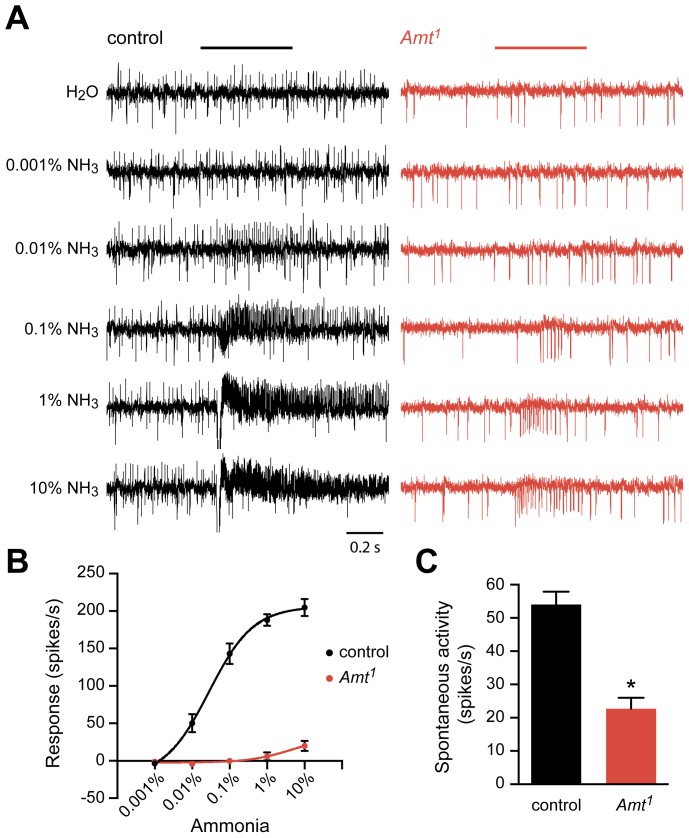
Reduced response of ac1 sensilla to ammonia in *Amt.* (A) Extracellular recordings of neuronal responses to a 500 ms pulse of either water or ammonia from ac1 sensilla of control and *Amt^1^*. ac1 sensilla were identified by their location and response characteristics (see Materials and Methods). Ammonia responses were quantified by subtracting the spike frequency elicited by water (solvent) from that elicited by ammonia and are plotted in (B). (C) The spontaneous spiking activity in ac1 sensilla was significantly lower in *Amt^1^* mutant sensilla compared to controls (n = 9 each, p<0.0001).

A key goal of our screen was to identify genes required for olfactory signaling in coeloconic sensilla. To ask whether *Amt* has a functional role in ammonia detection in ac1 sensilla, we tested an available mutant in which a transposon is inserted into the coding region of *Amt* [72]. We used single-unit electrophysiological recordings to test ammonia response at a wide range of doses. We quantified the number of spikes generated during a 500 ms odor stimulus period, and we subtracted the response to the odor diluent alone. Due to the difficulty of sorting spikes in ac1 sensilla, we quantified the total number of spikes from the three neurons in the sensillum, rather than the number of spikes generated by the ammonia-sensitive ORN alone. Control flies responded to increasing doses of ammonia with increasing numbers of action potentials, yielding spike frequencies similar to those observed previously (Figure 5A, B) [5], [6].

The *Amt* mutant (*Amt^1^)* showed a dramatically reduced response to ammonia (Figure 5A, B). Little or no response was seen in ac1 sensilla at most doses, and only very weak responses were seen at the highest dose. A second phenotype was also observed: mutant ac1 sensilla showed reduced spontaneous activity levels (p<0.0001) (Figure 5A, C). Moreover, the mutant ac1 sensilla appeared to lack the upward-directed spikes observed in control ac1 sensilla (Figure 5A). Close examination of the responses of ac1 sensilla to low concentrations of ammonia in control flies suggested that the upward-spiking neuron is the neuron that responds to ammonia, although this could not be determined conclusively.

**Figure 6:**
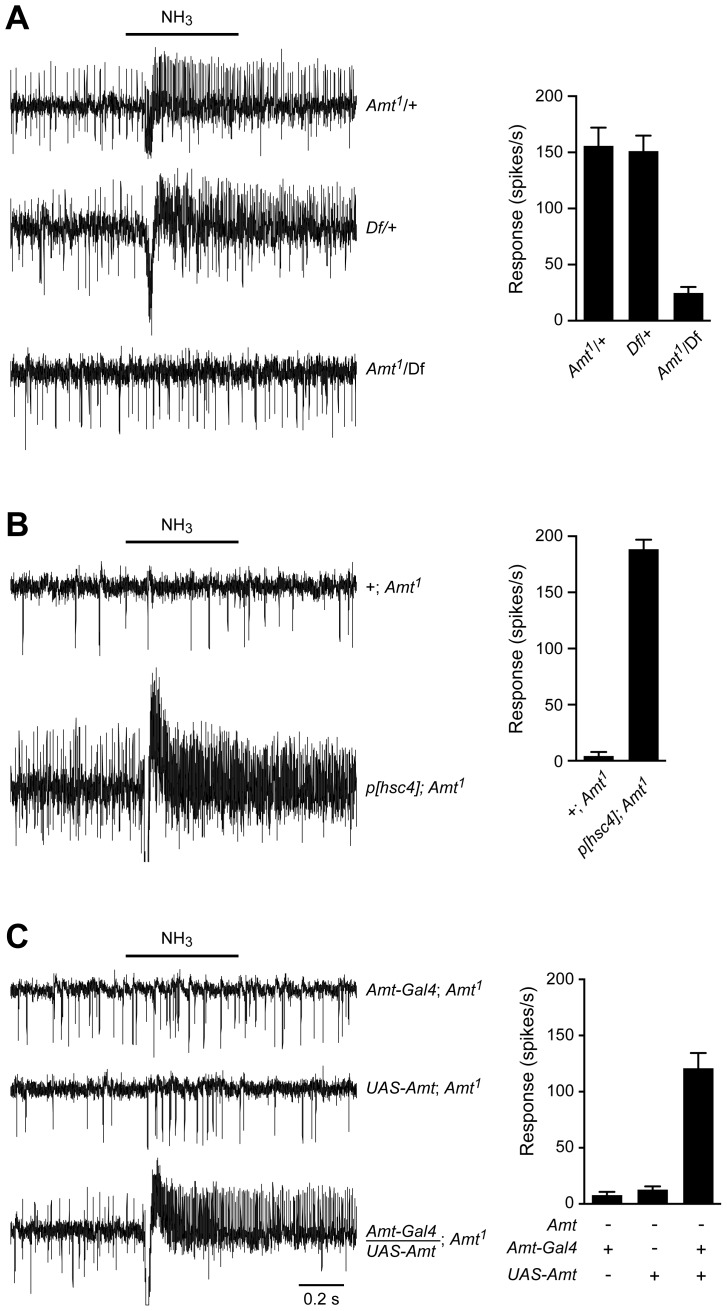
The loss of ammonia response localizes to the *Amt* gene. (A) Traces on the left show that the responses to a 500 ms pulse of 0.1% ammonia are similar in flies heterozygous for the *Amt^1^* transposon and the *Df(3R)BSC471* deficiency that removes ∼30 kb including *Amt* and nine other genes. In contrast, *Amt^1^*/*Df(3R)BSC471* flies have greatly reduced responses to ammonia (n = 9 each, p<0.0001). Averaged data are shown in the graph on the right. (B) The lack of response to 0.1% ammonia in ac1 sensilla from *Amt^1^* flies is rescued by the addition of a genomic fragment containing *Amt* and the neighboring gene *Hsc70-4* (n = 8 each, p<0.0001). (C) The response to ammonia in *Amt^1^* mutant flies is also rescued by transgenic expression of *UAS-Amt* under the control of an *Amt-Gal4* promoter (n = 9 each, p<0.0001).

We were surprised that a non-neuronal gene had such a dramatic effect on a neuronal response and sought to verify that the loss of ammonia response was in fact due to disruption of the *Amt* gene. The *Amt* mutant used in Figure 5B had been outcrossed for 10 generations to the control line so as to minimize genetic background effects, but we carried out additional tests. We sequenced the linked *IR92a* gene in both control and outcrossed mutant lines and confirmed they were the same. We then obtained a genetic deficiency (*Df*) that removed the *Amt* gene [73] and found that D*f/Amt^1^* heterozygotes showed a defective ammonia response (Figure 6A). Next we found that an available 14 kb genomic fragment including the entire coding region of *Amt,* as well as the neighboring gene *Hsc70-4* [74], rescued the defective ammonia response of *Amt^1^* (Figure 6B). Finally, we generated an *Amt* cDNA and showed that when driven by *Amt-GAL4*, the *UAS*-*Amt* cDNA construct rescued the *Amt* phenotype as well (Figure 6C).

### The *Amt* olfactory defect is specific to ac1 ammonia-sensitive ORNs

Given the unexpected severity of the *Amt* defect and the expression of *Amt* in auxiliary cells, we wondered whether *Amt* had a general effect on the function of the ac1 sensillum. For example, in addition to producing OBPs and ODEs, auxiliary cells generate the transepithelial potential, which is the electrical potential that underlies odor-induced ORN spiking [75]. To ask whether the *Amt* mutation affects the transepithelial potential or other general properties of ac1, we measured the responses of the other two ORNs in the ac1 sensillum.

**Figure 7:**
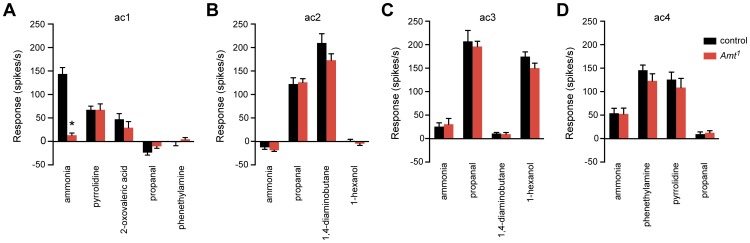
The *Amt^1^* defect is restricted to the ammonia response of ac1 sensilla. (A) Odor responses of ac1 sensilla in *Amt^1^*. Ammonia, 2-oxovaleric acid, and pyrrolidine each activate one of the three ORNs in ac1; only the ammonia response was impaired (n = 10–11, p<0.0001). (B–D) Response profiles of other sensillar types appeared normal (n = 8–9 each).

We tested pyrrolidine, which in wild type activates one of the other ORNs in ac1, and 2-oxovaleric acid, which activates the other ORN [5]. Both of these odorants elicited normal responses in *Amt^1^* (Figure 7A). These results suggest that there is neither a defect in the transepithelial potential of ac1 sensilla nor another general sensillar impairment, but rather a specific defect in ammonia response. In this experiment we also tested propanal and phenethylamine, odorants that in wild type distinguish ac1 from ac2, ac3, and ac4 [5], [6], and the responses to these odorants were characteristic of ac1.

We next asked whether *Amt^1^* affected olfactory signaling in the other three classes of coeloconic sensilla on the antennal surface, ac2–4. We tested odorants that activate different ORNs within each sensillum type, as well as negative control odorants selected to confirm the identity of each type. All responses were normal, consistent with the lack of *Amt* expression in these other sensilla (Figure 7B–D). These results indicate that the role of *Amt* is specific to ac1, which is particularly intriguing since ac1 is not the only sensillum that detects ammonia: ac4, for example, gives a weak ammonia response that does not depend on *Amt* (Figure 7D).

### An ortholog of *Amt* from an ammonia-seeking mosquito rescues the fly defect

**Figure 8:**
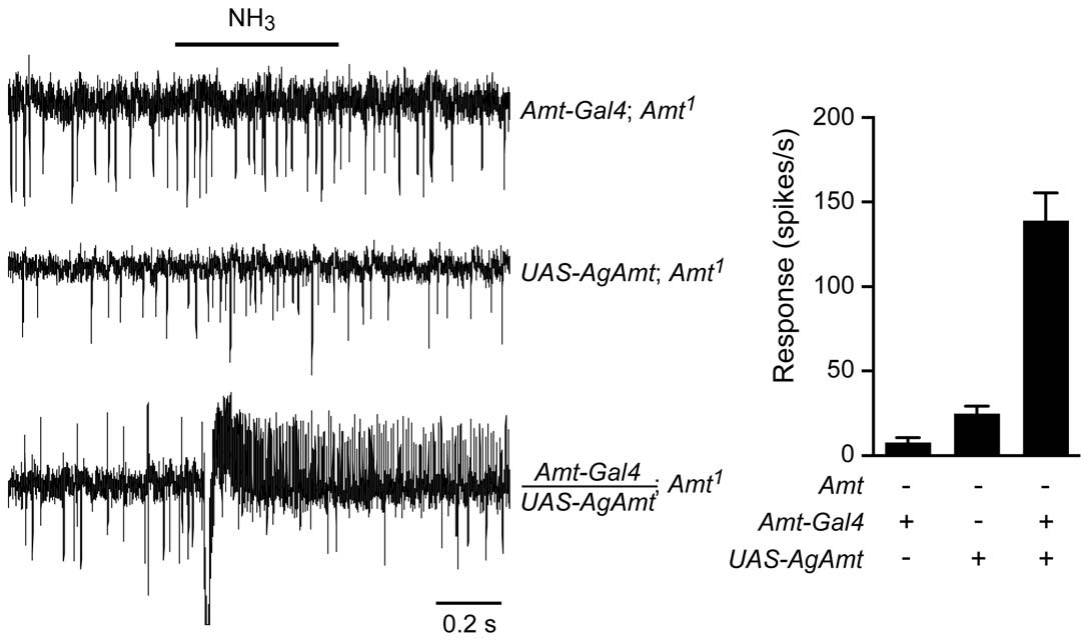
*AgAmt* from the mosquito *Anopheles gambiae* can substitute for *Drosophila Amt.* Traces on the left show that the lack of response to a 500 ms puff of 0.1% ammonia in *Amt^1^* mutant flies was restored by transgenic expression of *AgAmt* (n = 8 each, p<0.0001). Mean responses are shown in the graph on the right. Data for *Amt-GAL4; Amt^1^* are from Figure 6C.

Given the importance of ammonia detection in the process by which mosquitoes locate their human hosts, we cloned an *Amt* ortholog from the malaria mosquito *Anopheles gambiae* and generated a *UAS-AgAmt* fly line. We asked whether the mosquito construct could rescue the *Amt* defect in *Drosophila*. We found that the construct produced a robust rescue of the ammonia response (Figure 8), comparable in magnitude to that produced by the corresponding fly construct, *UAS-Amt* (Figure 6C). These results suggest that the two proteins have conserved function, consistent with their similar sequences.

## Discussion

This study has: i) provided a quantitative and comprehensive analysis of gene expression in the principal olfactory organ of *Drosophila*; ii) identified 250 genes in a screen for components of an evolutionarily ancient class of sensilla that are poorly understood but critical to insect olfaction; iii) shown that a transporter, despite its expression in non-neuronal cells, is essential for the response of a neuron to ammonia, a key cue for insect vectors of disease.

### Unanticipated expression of chemosensory receptors in the antenna

We detected expression of ∼8–10 *Gr* taste receptor genes that had not been previously found to be expressed in the antenna by RT-PCR or *in situ* hybridization. One, *Gr64f*, is expressed at particularly high levels. For nearly all of these genes we found spliced reads, confirming that the reads represent *bona fide* transcripts as opposed to genomic DNA. Moreover, the expression of all of these *Grs* in the antenna has been confirmed by qRT-PCR. They are unlikely to be expressed primarily in coeloconic sensilla, since their expression levels are not reduced in *ato* mutants. Further studies will be needed to localize the expression of these genes at higher resolution.

What roles might *Gr* genes play in the antenna? Most of these Grs are believed to act as receptors for sugars or bitter compounds, which have low volatility and are unlikely to reach the antenna via airborne transmission. In principle these antennal Grs could sense liquid or solid substances that make contact with the antenna, and perhaps then activate a grooming reflex that would clear foreign material. Alternatively, they could act as odorant receptors. Supporting this possibility, ab1D ORNs, which express *Or10a* and *Gr10a,* retain weak responses to some odorants in an *Or10a* mutant [76], and these responses could be mediated by *Gr10a.* As a third possibility, we note that the cytological localization of these Grs has not been determined, and these *Grs* might monitor internal metabolite levels, perhaps in compartments other than the sensillum lymph, as has been found for Gr43a in the brain [31]. Finally we note the possibility that these Grs may form heterodimers with Ors and modulate their ligand sensitivities or responses.

The RNA-Seq analysis detected transcripts of three olfactory receptors, *Or46a*, *Or33c*, and *IR60a*, whose expression in the antenna has not been observed with *in situ* hybridization. Our results are consistent with prior RT-PCR results [14], [25] and invite mapping of these receptors to sensilla and neurons of the antenna. Given that *IR60a* expression is reduced in *ato* flies, it is most likely expressed in either coeloconic sensilla or sensilla of the sacculus. In contrast, *Or46a* and *Or33c* expression is retained in *ato* mutants, and thus they are likely to be expressed in either basiconic or trichoid sensilla. Interestingly, both of these genes are expressed in the maxillary palp in an atypical manner. *Or46a* is alternatively spliced in the maxillary palp to encode three predicted receptors [77]. In the antenna we find sequences corresponding to only one receptor, Or46aB (Dataset S1). *Or33c* is unusual in that it is coexpressed in a maxillary palp ORN with another *Or* gene, *Or85e*, and this coexpression has been conserved for >25 million years [26]. It will be interesting to determine if the *Or46a* transcript maps to the ab6B neuron, whose response profile bears similarity to that of pb2B, a neuron that expresses another splice form of *Or46a* [8], [9], [77].

Our RNA-seq analysis of wild type antennae is consistent with a recently published transcriptional profiling analysis of the antenna by Shiao *et al.* [78], in the sense that chemoreceptors with relatively high expression levels in our analysis were also found at relatively high levels by Shiao *et al.* Furthermore, the unexpected chemosensory receptors that we identified were also detected by Shiao *et al.* However, the analysis by Shiao *et al.* detected the expression of a much greater number of chemoreceptors in the antenna than we did: 50 *Ors* and 35 *Grs*. These greater numbers may result at least in part from the use of relatively relaxed criteria for expression and a lack of biological replicates. We note that the 50 *Ors* include a substantial number that were previously found to be expressed only in the maxillary palp or in larvae in studies based on *in situ* hybridization or reporter gene expression analysis [10], [11], [14], [15], [24], [25].

We were surprised by the wide dynamic range of expression within gene families. Levels of *OBP* transcripts, for example, varied over several orders of magnitude. It is possible that these RNA levels do not faithfully represent protein levels. However, we note that most of the OBP transcripts that were most abundant in our RNA-Seq analysis correspond to OBP proteins that were among the most abundant in a proteomics analysis [79]. Canonical Or transcripts varied over a 170-fold range, and it would be of special interest to examine the levels and localization of tagged Or proteins representing *Or* genes expressed at both high and low levels.

### Genes that distinguish coeloconic sensilla

Other than *Or* and *IR* genes, few genes had previously been found to distinguish coeloconic sensilla from other sensillar classes, despite their ancient evolutionary origins, different odor response spectra, and distinct morphology. Our screen identified 250 antennal genes that are reduced in expression levels by at least 4-fold in *ato* antennae, which lack coeloconic sensilla.

Of the 10 most significantly reduced genes, four have been implicated in odor coding in coeloconic sensilla, and another is an OBP. None of the 10 most highly ranked genes are expressed broadly or at high levels outside of the head, and none have been associated with gross abnormalities in mutant screens. It thus seems likely that many of the genes identified in our differential screen contribute specifically to the function of coeloconic sensilla.

Of these 250 genes, many may act to direct or modulate neuronal activity: the set of candidate genes is highly enriched in ion channels, receptors, and transporters. The set also contains many biotransformation enzymes, which detoxify xenobiotic molecules and are of special interest because they contribute to insecticide resistance [80]. The olfactory organs of both vertebrates and insects, including *Drosophila,* express biotransformation enzymes at high levels [21], [81]–[85], perhaps because these organs are directly exposed to xenobiotic molecules in their environment. Some of these enzymes have been shown to degrade odorants *in vitro* [21], [81], [86]. Our results suggest that some biotransformation enzymes may be specialized for the degradation of odorants in coeloconic sensilla. Although airborne odorants presumably have access to all sensilla of the antenna, the concentration of certain odorants may be tightly regulated in some sensilla, particularly in the sensilla that contain responding ORNs.

Other members of the set of 250 genes encode transcription factors, cytoskeletal proteins, and proteases that may contribute to the establishment and maintenance of the signaling environment in these sensilla. A large fraction of genes, one-third, are of unknown function. Taken together, our results suggest that coeloconic sensilla contain many diverse components that are distinct from those of other sensillar classes, likely reflecting their different evolutionary origin and their distinct olfactory function.

### An ammonium transporter critical for ammonia responses of ac1 coeloconic ORNs

We found that a transporter, Amt, is essential to olfactory signaling. Ammonia transport across biological membranes is a critical feature of nitrogen metabolism across phylogeny. Members of the conserved Amt family [87], [88] transport ammonia in bacteria, archaea, fungi, plants, and invertebrates, but not vertebrates [64]. Many microorganisms and plants use Amt transporters to acquire ammonia from their environment [87], [88]; they then metabolize the ammonia into amino acids and other molecules. The related Rh ammonium transporters, members of the SLC42 solute transporter family, act in the excretion of ammonia in vertebrate kidneys [89], [90] and may play a similar role in the orthologous Malpighian tubules of insects [91], [92]. Ours is the first report of a role in olfactory signaling for an ammonium transporter.

We found that the *Anopheles gambiae* homolog AgAmt can substitute for *Drosophila* Amt in an Amt mutant background. The vast majority of insect genomes examined contain a single *Amt* gene [93], and *Amt* transcripts are found not only in mosquito antennae [83], [94] but also in EST collections from a variety of insect pests, including the cotton leafworm, the emerald ash borer, and the Old World bollworm [82], [95], [96]. Taken together, these results suggest a conserved role in insect olfaction. AgAmt could be a useful target for disrupting ammonia-based chemoattraction of mosquitoes to humans, especially since Amt homologs are not present in humans or other vertebrates.

In *Drosophila*, the only Amt expression we detected in the antenna outside of ac1 sensilla was in the third chamber of the sacculus. The sensilla of this chamber are not accessible to electrophysiological recordings, but GCaMP imaging has indicated that ammonia in fact elicits a response from some of these sensilla, although interestingly the response was an inhibitory one [97].

Interestingly, Amt is not required for the weak ammonia responses of ORNs in ac3 and ac4 sensilla. It is possible that ammonia is transported in these sensilla by Rh50, a member of the family of Rh ammonium transporters. Rh50 is found at low levels (∼21 RPKM) in the antenna of wild type flies, and its expression is ∼7-fold reduced in *ato*, suggesting that it is expressed in coeloconic sensilla (Dataset S1). We note that when the ac1 ammonia receptor IR92a was ectopically expressed in ac4 ORNs, it did not confer the strong ammonia response characteristic of ac1 [15] (but see [69]). It was proposed that the lack of a strong ammonia response was due to the lack of a factor that is present in ac1 but not ac4. Amt could be such a factor.

The simplest mechanistic explanation for how Amt acts is that it plays a role in clearing ammonia from the sensillar lymph (Figure S8). *Drosophila* larval haemolymph contains ∼1 mM ammonia [98], derived from the animal's internal metabolism. Sensillar lymph may also contain ammonia from the fly's metabolism, and in addition is exposed to volatile ammonia from the fly's environment. Ammonium levels in *Drosophila* culture vials have been measured at ∼20–30 mM [98], [99], primarily from the microbial ammonification of waste products. Although a low concentration of ammonia in sensillar lymph may be inconsequential to the function of most sensilla, it may interfere with the sensitive detection of ammonia by the IR92a-expressing ORN in ac1 sensilla. Amt may be necessary, in non-cell autonomous fashion, to lower basal concentrations of ammonia in ac1 to allow this ORN to detect transient volatile ammonia stimuli in the fly's environment (Figure S8A). In an Amt mutant, the ammonia concentration in the lymph would be higher than in wild type and could lead to tonic adaptation of IR92a or the ORNs that express it (Figure S8B). There is precedent for such adaptation. Low micromolar concentrations of agonist desensitize some types of ionotropic receptors, including vertebrate glutamate receptors that are related to the IRs [100], and inhibition of a GABA transporter leads to reduced GABA-mediated currents at a vertebrate synapse due to such desensitization [101]. Thus, tonic adaptation could explain the inability of the Amt mutant to respond to ammonia, except at the highest concentration tested, and may also explain the low spontaneous firing rate observed in sensilla of the AMT mutant.

### Auxiliary cells and their contributions to an olfactory circuit

Many studies of sensory coding consider the neural circuit in isolation. In olfaction, most research has focused on the responses of ORNs and how the odor representations produced by ORNs are successively transformed at higher levels of the olfactory circuit. However, sensory neurons function in complex tissues that can influence their activity profoundly.

Auxiliary cells surrounding insect ORNs produce two classes of proteins that are thought to contribute to olfactory coding: OBPs and ODEs. These proteins have been identified in many insects, their expression has been studied, and in many cases they have been shown to bind or degrade odorants *in vitro* [20], [21]. Genetic demonstrations of their roles in olfaction *in vivo* have been sparse; however, in *Drosophila,* mutants of the OBP Lush show reduced responses of trichoid sensilla to the pheromone 11-*cis*-vaccenyl acetate [45], and mutants of the ODE Est-6 show prolonged decay kinetics in response to this pheromone [102].

Our study demonstrates *in vivo* a role for a third class of protein in auxiliary cells: transporters. We have shown through genetic analysis that a transporter, *Amt*, makes a profound contribution to ammonia sensing by ac1 ORNs. Our transcriptional profile of the antenna identified many genes that are described by the term “transporter activity”, many of which were reduced more than 4-fold in *ato* mutants. The *Drosophila* olfactory system encodes a remarkable number of structurally diverse odorants. It will be interesting to determine whether perception of this rich diversity of odorants depends on a wide diversity of transporters.

## Materials and Methods

### *Drosophila* stocks

Canton-S and *atonal* (*ato^1^/Df(3R)p^13^*) [58] flies were used for RNA sequencing experiments, and Canton-S flies were used for RT-PCR and qRT-PCR. *GAL4* drivers, either generated as described below or gifts from R. Benton [5], [15], were crossed to a *UAS-mCD8::GFP* line [103]. The *Amt^1^* transposon insertion line, *P{EPgy2}CG6499[EY21789]* (Bloomington Drosophila Stock Center) [72], was outcrossed for ten generations to an isogenized *w^1118^* line [104]; this outcrossing removed a nearby lethal mutation. The outcrossed *Amt^1^* line and the isogenic control line were used for electrophysiology experiments. As indicated in the text, the outcrossed *Amt^1^* line was also crossed to a deficiency line, *Df(3R)BSC471* (Bloomington Drosophila Stock Center) [73], a genomic rescue line *p[hsc4]* (Spyros Artavanis-Tsakonas) [74], or to *GAL4* and *UAS* lines generated as described below.

### Transgenic fly generation

To generate *GAL4* transgenes, 5′ and 3′ flanking regions were amplified using PfuUltra II Fusion HS DNA Polymerase (Agilent Technologies) from tiling bacterial artificial chromosomes (BACs) corresponding to the reference *Drosophila melanogaster* genome. 5′ and 3′ flanking regions and *GAL4* were cloned in pDONR vectors, and then assembled into the pBGRY destination vector via MultiSite Gateway Pro 3-fragment recombination (Invitrogen). pBGRY, derived from pBPGUw [105], contains a phiC31 attB site, the mini-*white* gene, and a pair of *Su(Hw)* insulator elements [106].

*Amt-GAL4:* The 5′ fragment extended from 11077118 to 11074858 and the 3′ fragment from 11072987 to 11072168 on chromosome 3R.

*IR76b-GAL4:* The 5′ fragment included 20105430 to 20104538 and the 3′ fragment included 20101957 to 20096450 of chromosome 3L.

*IR8a*-*GAL4:* The 5′ fragment spanned from 9131297 to 9130620 and the 3′ fragment from 9126786 to 9126175 of chromosome X.

Antennal cDNA from Canton-S flies and *Anopheles gambiae* mosquitoes was used to generate *UAS-Amt* and *UAS-AgAmt* transgenes respectively. The full-length open reading frames (ORFs) of *Drosophila CG6499/Amt* (accession number NM_001104330.3) and *Anopheles AGAP003989* (accession number XM_318439.3) were amplified with a CAAC Kozak consensus sequence upstream of the start codon and cloned into pDONR vectors. These vectors were recombined into the pBID-UASC-G destination vector [107] using Gateway Cloning (Invitrogen).

Assembled *GAL4* and *UAS* vectors were used to generate *Drosophila* strains through PhiC31 integration into either attP40 (second chromosome, *Amt-Gal4*, *UAS-Amt*, and *UAS-AgAmt*) or attP2 (third chromosome, *IR76b-Gal4* and *IR8a-Gal4*) landing sites (BestGene Inc.) [108], [109].

### RNA isolation and sequencing

The third antennal segments from ∼300 adult Canton-S and *ato* flies aged 5–12 days after eclosion were carefully hand-dissected from the head, and fell immediately into 1.5 ml microfuge tubes kept cold in liquid nitrogen. Three independent biological replicates were collected per genotype, ∼900 total flies. Antennae were mechanically crushed with disposable RNAse-free plastic pestles and a QIAshredder column (QIAGEN), and total RNA was harvested using an RNeasy Mini Kit (QIAGEN). Total RNA (∼0.5 µg/sample) was provided to the Yale Keck Biotechnology Resource Laboratory. There, polyA^+^ RNA was selected and fragmented, and samples were prepared for single-end mRNA sequencing (RNA-Seq) with standard protocols, and cDNA libraries from each sample were sequenced with an Illumina Genome Analyzer II running pipeline version 1.3.2 to generate 37 bp reads. Total reads reported were those that passed the associated quality controls.

### RNA-Seq data processing and analysis

Illumina's ELAND program was used to align trimmed 35 bp reads to the Drosophila reference genome (BDGP Release 5), omitting ArmUextra as this consists of degenerate sequences not incorporated into the genome assembly [110]. The program was configured to allow up to two mismatches per read. Reads that did not align to the genome were also aligned to an mRNA splice junction set (see below, Dataset S1, “by splice”). Per sample, 84–92% of total reads were uniquely aligned to either the genome or the splice junction set, for a total of ∼4.3 to 7.6 million aligned reads per sample (Table S1).

The 14,078 non-redundant annotated genes in FlyBase Genes release 5.12 (Oct. 2008) were used for gene expression analysis using Illumina's CASAVA program (version 1.0). This gene annotation dataset was also used to generate the splice junction set used for read alignment. Aligned reads were mapped to gene exons based on their genomic positions (Dataset S1, “by exon”). Genome positions associated with multiple genes in the annotation set were considered ambiguous and not analyzed. The exon and splice junction reads mapped to a given gene were summed to determine its expression (Dataset S1, “by gene”). Each gene's expression was then normalized by the total mapped reads in that lane to generate the reads per million reads mapped (RPM) value. Gene expression similarity between samples was visualized with hierarchical cluster analysis of all 14,078 genes using Ward's method using a function in R (version 3.0.1) [111]. Genes were defined as “CS-expressed” or “*ato*-expressed” if they were detected at >1 RPM in all three samples from the same genotype. Gene expression was further normalized by its gene length to generate the Reads Per Kilobase per Million reads mapped (RPKM) value and averaged by genotype (n = 3 each, CS and *ato*, Dataset S1, “by gene”).

We note that the standard gene symbols for some genes changed after our initial data collection and analysis. Thus the original symbols for the mapped genes are found in Dataset S1, but we have also included the current gene symbols and FBgn numbers in the “by gene” and “differential expression” worksheets. For some genes, exon structures have been reannotated and some exons reassigned since our original data collection and analysis, and thus there are likely to be some small discrepancies between the annotations used in Dataset S1 and current annotations. Additionally, some genes have been withdrawn from the current annotation set, but are still listed in Dataset S1. The current gene names are used in the main text and figures.

### Differential expression analysis

Statistical analysis of differential gene expression was carried out with the EdgeR package (3.2.3) [112]. Differential expression analysis was performed on the 9,034 genes detected at >1 RPM in at least 3 of the 6 total samples. EdgeR uses the raw read counts for each gene and normalizes each sample for differences in sequencing depth and compositional bias. Dispersion is estimated using the quantile-adjusted conditional maximum likelihood method, and differential gene expression between groups (here, CS versus *ato)* is tested using an exact test, based on read counts obeying a negative binomial distribution [112]. See the EdgeR User's Guide for further details (www.bioconductor.org). The p-value and false discovery rate (FDR) for each gene are reported in Dataset S1, “differential expression”. We considered genes to be differentially expressed if they had a FDR <0.01. The average expression across all six samples, log_2_(RPM), and the fold-change, log_2_(RPM*_ato-_*_avg_ */*RPM_CS-avg_), both based on the EdgeR normalized samples, is also reported in Dataset S1, “differential expression”.

We note that EdgeR moderates each of the raw read counts by adding a very small value (prior.count) to each one, thereby avoiding undefined fold-changes such as when one group has zero reads (for example log_2_(0/RPM_CS-avg_)). This process creates the diagonal “line” of red dots seen in Figure 2C, a plot of log_2_(RPM) versus log_2_(RPM*_ato-_*_avg_ */*RPM_CS-avg_). An explanation of why this occurs can be seen in the following example for genes with zero reads in all three *ato* fly samples and at least a moderate number of reads in CS flies:

1. The average expression across all samples, RPM is equal to (RPM_CS-avg_ + RPM*_ato_*_-avg_)/2. After addition of prior.count to each sample, RPM*_ato_*_-avg_ is still approximately 0 and much less than RPM_CS-avg_. Therefore RPM is close to (RPM_CS-avg_)/2.
2. The x-value in Figure 2C for these genes is thus log_2_(RPM_CS-avg_/2)  =  log_2_(RPM_CS-avg_) - 1
3. The log fold-change log_2_(RPM*_ato-_*_avg_ */*RPM_CS-avg_)  =  log_2_(RPM*_ato-_*_avg_) - log_2_(RPM_CS-avg_). For all genes with zero reads in *ato* samples, RPM*_ato-_*_avg_ will be a fixed constant c based on the prior.count added to each sample.
4. The y-value in Figure 2C, log fold-change, for these genes is thus c - log_2_(RPM_CS-avg_).
5. For these genes, their x-values are thus log_2_(RPM_CS-avg_)-1 and their *y*-values are c- log_2_(RPM_CS-avg_). Through substitution, the y-values can also be expressed as y =  c-1-x, and thus the dots in Figure 2C will fall along the line as a function of their x-value.

### Functional annotation of genes

Protein sequences were manually curated using FlyBase (http://www.flybase.org), BLAST (http://www.ncbi.nlm.nih.gov/BLAST), and SMART (http://smart.embl-heidelberg.de). The percentage of level 2 Gene Ontology (GO) terms in each dataset and significantly enriched GO terms were identified using AmiGO version 1.8, GO database release 2013.08.17 [113]. Some gene symbols in the *ato*-depleted and *ato*-enriched datasets were renamed according to current gene annotations in order for AmiGO to recognize the gene symbols. Accordingly, the *ato*-depleted gene *CG14586* was divided into *IR75b* and *IR75c*, and *CG34372* and *CG5357* were combined into *CG43795*.

### RT-PCR and quantitative real-time PCR (qRT-PCR)

Tissues from male and female flies aged 5–14 days and 3^rd^ instar larvae were collected for RNA extraction. Heads (∼25), heads with antennae removed (∼25), bodies with legs and heads removed (∼25), antennae (∼200), legs (∼600–900) or whole larvae (∼25) were dissected using forceps and placed immediately into 1.5 ml microfuge tubes kept cold in liquid nitrogen. Tissues were crushed with plastic pestles and a QIAshredder column (QIAGEN), and RNA was harvested using an RNeasy Mini Kit (QIAGEN). An iScript cDNA Synthesis Kit (Bio-Rad) was used to generate cDNA. To control for contamination by genomic DNA, each RNA sample also underwent a parallel mock reverse transcription step in which the reverse transcriptase was omitted.

RT-PCR expression analysis was carried out with GoTaq Flexi (Promega) and a 30 cycle PCR. *Amt* expression was assayed with primers: CGGTGTTCAGGAAGGAGAAC and TTCCCGGTCTGTATGACCTC. To control for cDNA quality, the presence of synaptotagmin I in each sample was assayed with the primers CGGATCCCTATGTCAAGGTG and TCTGGTCGTGCTTCGAGAAG. *IR25a* expression was assayed with the primers CAATCCACTCAGCCATTCAA and AGTGGACAATTGCGGCTATC. *IR8a* expression was assayed with the primers GCTGGAGTGGCATTTCGTAT and GGTAGATGGCCAACGGATAA. All primer sets spanned an intron to distinguish cDNA from genomic DNA.

CS cDNA obtained as described above was used for qRT-PCR in a BioRad iQ5 machine. Each 18 µl reaction containing cDNA generated from 2.5 ng RNA, 1× SsoFast EvaGreen Supermix (Bio-Rad) and 400 nM primers was run in triplicate according to the Supermix protocol. Expression of each gene was normalized to eIF-1A. Transcript-specific *Gr* primers were designed with Primer-BLAST (NCBI) such that they spanned an exon and amplified 150-250 bp fragments. Negative controls without reverse transcriptase and without RNA were run to exclude genomic contamination and primer-dimer formation. Each reaction was run on 3–4 independent sets of CS antennae.

Primers used were:

eIF-1A: ATCAGCTCCGAGGATGACGC and GCCGAGACAGACGTTCCAGA

Gr64a: AGCCAAGAATTTTGTGGGATTGG and GCATGTTCGCCTAAGGACAAG

Gr64b: CTGGAGCACCTCTTCTTCTGG and ACAGTGTTCCACCAAAGCTG

Gr64c: AATGCAGATGCGATTCCAGC and GTTGCCCTTGGATTGAAAGC

Gr64d: ACCATAGTTTTCAGGTCAAAGGA and AAACGACCCAGTTCATCGCA

Gr64e: AAGCCATCAAGCCTGTCCTC and CAGGGTCTCCACCGAATCAA

Gr64f: GGCGGTTTCACTGTACTCCTC and ATGGTTCCAGCCACACTCAG

Gr43a: CCCGAGAGTCCCGTAAAACG and GCGGATGCAAACGATGTCAG

Gr66a: AATTCTGCCACAGGATCTCG and CGAAAGTCAAGGTGCTCTGC

Gr61a: CTGGAGGGTCGTCATGTTCC and GGTGAAAATAGCCAACGCCTG

Gr93a: CGATGGGATAAGAGTGTTGAAAC and CCACCTGTAATGCCGAACTG

Gr10a: GGCTGACCAGGGAGATAGAAC and AGAGATCGAACTGCACCAGAG

Gr63a: AAGCCGAGTGTTTTCTACCG and CCTACATAGCACGCCAGG

Gr21a: TCTACCCACTCACCTGGTCG and TTGCAGTTGATGTACCACAAGC

Gr28b: ACATTGTATTTCACGATCAGCG and CCTTCGATTTCCATCCCCCAT

### Whole-mount imaging

7 day old male and female *attP40{Amt-Gal4}; UAS-mCD8::GFP* flies were frozen with liquid nitrogen and their antennae dissected into a solution of 0.5× PBS, 0.1% Tween-20, and 50% glycerol. After soaking for at least 5 minutes, antennae were transferred to glass slides and imaged within a few hours. Confocal stacks were acquired at 40× on a Zeiss LSM510 confocal microscope and processed using NIH ImageJ (version 1.44o).

### RNA probe synthesis for *in situ* hybridization

The *Amt* coding region was amplified from CS antennal cDNA and cloned into the pGEM-T Easy vector (Invitrogen) for transcription. Digoxigenin (DIG) labeled probes for *Amt* were created using the DIG RNA Labeling Kit (SP6/T7) (Roche) and hydrolyzed for 1 hour in 30 mM Na_2_CO_3_, 20 mM NaHCO_3_ (pH 10.2). The reaction was stopped with 3 M NaOAc, 1% acetic acid (pH6), and then the probe was purified via ethanol precipitation, solubilized in DEPC H_2_O, and stored at -80°C. The mCD8::GFP probe was created similarly but from the pBS mCD8::GFP plasmid [103] using T3 polymerase and Fluorescein (FITC) labeled UTP (Roche). The *Obp84a* FITC probe was created similarly, but was not hydrolyzed, and was instead purified with the RNEasy Cleanup Kit (QIAGEN).

### *In situ* hybridization and immunohistochemistry

7 day old flies were anesthetized, placed in a collar, covered with OCT (Tissue-Tek), and frozen on dry ice. 14 µm antennal sections were collected on slides and fixed for 10 minutes in 4% formaldehyde in PBS. All steps were performed at room temperature unless otherwise noted. Sections were washed 3×3 minutes in PBS, acetylated for 10 minutes (0.925 g triethanolamine HCl, 112 µl NaOH, and 125 µl acetic anhydride in 50 ml DEPC H_2_O), washed 3×5 minutes in PBS, and prehybridized for 1 hour at 65°C in Hybridization Buffer (HB) (50% formamide, 5× SSC, 50 µg/ml heparin, and 0.1% Tween-20). DIG and/or FITC labeled probes were diluted to 500 ng/ml in HB and applied to sections, which were then covered with hybrislips (Sigma-Aldrich) and incubated in a humidified chamber for 18–20 hours at 65°C. Hybrislips were removed by soaking slides in 5× SSC at 65°C, then sections were washed 3×20 minutes in 0.2× SSC at 65°C. Sections were incubated for 10 minutes in TN (100 mM Tris-HCl, pH 7.5, 150 mM NaCl), then for 30 min in TNB (TN plus 1% Blocking Reagent (Roche)). Anti-DIG-POD (Roche) was diluted 1∶500 in TNB and applied to slides for 30 minutes. After washing with agitation 3×5 minutes in TNT (TN plus 0.05% Tween-20), sections were incubated with Cy3-tyramide diluted 1∶50 in amplification reagent (TSA kit, Perkin Elmer) for 10 minutes. For double-label *in situ* hybridizations, sections were then washed with agitation 3×5 minutes in TNT, incubated for 30 minutes in 3% H_2_O_2_-TNT, washed with agitation 3×5 minutes in TNT, blocked for 30 minutes in TNB, incubated for 30 min with anti-FITC-POD (Roche) at 1∶500 in TNB, washed with agitation 3×5 minutes in TNT, and incubated with 1∶50 Cy5-tyramide in amplification reagent (TSA kit, Perkin Elmer). All sections were washed with agitation 3×5 minutes in PBST (PBS plus 0.1% Tween-20), blocked for 30 minutes in 1% BSA-PBST, then incubated overnight at 4°C with mouse anti-GFP (Roche) diluted 1∶500 in 1% BSA-PBST. Sections were washed 3×5 minutes in PBST, then incubated for 2 hours with Alexa Fluor 488 donkey anti-mouse antibody (Invitrogen) diluted 1∶500 in 1% BSA-PBST. Sections were washed 2×10 minutes in PBST and mounted in Vectashield. All microscopy was performed using a Zeiss LSM 510 Laser Scanning Confocal Microscope, and images were processed with ImageJ software.

### Electrophysiology

Female flies, 5 days after eclosion, were used for single-sensillum recordings essentially as described [114]. The stabilized antennae and heads of mounted flies were visualized with an Olympus BX51WI microscope. A glass capillary electrode filled with sensillum lymph ringer solution [115] was inserted into the base of a coeloconic sensillum, and a reference electrode filled with the same solution was placed in the eye. An Iso-DAM amplifier (World Precision Instruments) was used for extracellular recordings. Filtered AC signals (300–2,000 Hz) were collected and digitized at 5 kHz with a Digidata 1322A digitizer and Axoscope 9.2 software (Molecular Devices).

Action potentials were detected and counted offline in a 500 ms response period using AxoGraph × (version 1.31) software. Responses started ∼100 ms after the onset of the stimulus, presumably due to the time odors took to travel to the antennae, and the response period was therefore defined as beginning 100 ms after the beginning of the stimulus period. We summed all spikes from the 2–3 neurons in a given sensillum because of difficulties in sorting spikes in coeloconic sensilla [5], [15]. Responses of an individual sensillum to an odorant were calculated as the change in spike rate relative to its response to the relevant solvent (paraffin oil or water). Responses to solvents alone were generally negligible. Each sensillum was tested with multiple odorants, and no more than three sensilla were analyzed per fly. Spontaneous spikes were counted in the 500 ms prior to the stimulation period of the first odor. Coeloconic sensillar subtypes were found in characteristic regions on the antenna, and were definitively distinguished by their responses to a small set of diagnostic odors [5], [6], [15]. Although *Amt^1^* ac1 sensilla lacked a strong, characteristic response for positive identification, they could be reliably identified by their location, absence of responses to either propanal, which strongly activates ac2 and ac3, or phenethylamine, which strongly activates ac4, and their relatively low spontaneous firing rates.

Statistical significance was assessed with either a Student's t-test, one-way ANOVA, followed by Tukey's post-hoc test, or two-way repeated measures ANOVA followed by Bonferroni's post-hoc test, as appropriate. Values shown are the mean ± SEM.

### Odor stimuli

Ammonium hydroxide (28–30%, Sigma) was diluted in molecular biology grade water (Sigma) 1∶3 to generate 10% ammonia. Further serial dilutions were used to generate lower concentrations. Unless indicated otherwise, 0.1% ammonia was used for electrophysiology experiments. Other odorants were of the highest grade available (97% to >99%) and were used at the following dilutions (v/v) in either water: 0.1% 1,4-diaminobutane (Aldrich), 1% pyrrolidine (Fluka), or parrafin oil (Fluka): 1% propanal (Aldrich), 1% phenethylamine (Sigma), 0.001% 1-hexanol (Fluka), and 1% 2-oxovaleric acid (Fluka).

Odorant stimuli were prepared and delivered essentially as described previously [9], [114]. Odor stimuli cartridges were prepared by inserting a 0.5 inch diameter filter disk containing 50 µl of diluted odorant into a Pasteur pipette and capping the end with a 1 ml pipette tip. Cartridges were allowed to equilibrate for at least 20 minutes before use and were used no more than four times. Stimuli were presented by placing the tip of the cartridge through a hole in a glass tube carrying a humidified air stream (2,000 ml/min) directed at the fly and administering a 500 ms pulse of air (∼600 ml/min) through the cartridge.

## Supporting Information

Figure S1 Olfactory receptor gene expression in CS and *ato* flies. (A) The 39 *Ors* and (B) 19 *IRs* expressed in CS fly antennae are listed by descending expression level (RPKM), averaged across the three samples. Like other genes, the olfactory receptor genes are considered expressed in CS if there were at least 1 RPM in each of the three CS samples. For comparison, the average expression levels of the same olfactory receptors are provided for *ato* flies.
(TIFF)

Figure S2 Auditory organ gene expression in the third antennal segment. The 20 most highly expressed auditory organ genes are listed in descending order by their expression level (data from[27]). The expression of auditory organ genes was determined by averaging the mean microarray fluorescence intensities from the six “control” replicates of each of the 274 auditory organ genes listed in Table S2 of Senthilan et al. 2012. “+” indicates genes that were detected at >1 RPM in each of our three CS third antennal segment samples, *i.e.* genes that met our standard criteria for expression in CS. Seven of the 20 genes were not considered expressed, suggesting that the auditory organ of the antenna, the second antennal segment, did not substantially contaminate our collection of the olfactory third antennal segments.
(TIFF)

Figure S3 Olfactory receptor expression by sensillum type and ORN class. In *Drosophila*, olfactory sensilla house 1–4 ORNs that can be distinguished by their odor response profiles. Stereotyped groupings of ORNs form different sensilla types. Individual ORNs are designated by their sensillar morphology and type: large basiconic (ab1–3), small basiconic (ab4–10), trichoid (at1–4), or coeloconic (ac1–4) sensilla. Additionally, many ORNs are designated by the relative size of their spike amplitude, such that the A neuron has the largest amplitude spikes within a sensillum type, the B neuron has the next largest, etc. Previous studies have mapped individual olfactory receptors to specific sensilla types, and in many cases to particular ORNs [5], [6], [9]–[11], [15], [28]–[30], [76], [117], [118]. This table lists the average expression level of each olfactory receptor detected in CS flies by its sensillar type and ORN. In some sensillum types, specific ORNs have not been identified, and we use the suffixes X, Y, and Z to designate the different ORNs in order to clarify which receptors are co-expressed in individual ORNs. Different sensillum types are separated by a thin line. Some IRs are found in multiple coeloconic types and are listed more than once, and IRs found in the sacculus and artista are also listed. The broadly expressed co-receptors Orco, IR8a, IR25a and IR76b are not listed here.
(TIFF)

Figure S4 Chemosensory gene expression in CS and *ato* flies. (A) 12 *Gr* genes are expressed in CS antennae and are listed by descending expression level (RPKM) averaged across samples. Their expression levels in *ato* are also indicated. (B) CS-expressed Obps are detected at a wide range of expression, and a subset are substantially reduced in *ato* flies. We note that *Obp76a* is formally known as *lush*. (C) Most *Drosophila* Trp channel family members are detected in CS antennae, albeit at relatively low levels, and most are found at similar levels in *ato* flies.
(TIFF)

Figure S5 Validation of chemosensory gene expression. (A) Expression of 14 *Gr* genes was verified by qRT-PCR using antennal cDNA. Expression was quantified relative to a control gene amplified in all reactions, and the *Gr* genes are listed in the same order as in Figure S1F to facilitate comparison of relative levels of gene expression. *Gr64d* and *Gr64e* are also detected in the CS antenna by qRT-PCR and are listed last. (B) RT-PCR on CS antennal cDNA indicates that *IR8a* expression is lost in *ato* flies, whereas substantial expression of *IR25a* remains. Both genes are found in CS antennae.
(TIFF)

Figure S6 Genes most highly expressed in CS antennae. The ten genes with the highest expression levels in CS antennae are listed in decreasing order of expression level (by RPKM).
(TIFF)

Figure S7 Gene ontology terms enriched in CS and *ato* fly antennae. Gene ontology (GO) terms represent gene product properties in three categories: Cell component, Molecular function, and Biological process. These three descriptors are called “level 1” terms. Lower level terms are more specific and form a subset of the higher level terms. These subsets overlap, and thus a gene can be annotated with multiple different GO terms, such as “binding” and “receptor activity” within Molecular function. Many, but not all, individual genes have been either manually or automatically annotated with GO terms. In total, 13,737 Flybase *D. melanogaster* genes, 185 of 250 *ato-*depleted genes, and 130 of 155 *ato-*enriched genes have been annotated with at least one GO term. The graph depicts the percentage of the annotated genes in each group that are annotated with each level 2 GO term. In general, the percentage of genes annotated with each GO term was comparable between groups. However, the GO analysis program AmiGO detected a significantly higher proportion of the 185 *ato*-depleted genes associated with the GO terms “membrane”, “transporter activity”, “molecular transducer activity”, and “receptor activity” compared to the FlyBase genes (see also Figure 3). Significant enrichment of the terms “extracellular region”, multi-organism process”, and “immune system process” were detected in the 130 *ato-*enriched genes compared to the FlyBase genes. For simplicity, level 2 GO terms were only included if at least 1% of genes in one of the three groups are annotated with a given GO term.
(TIFF)

Figure S8 A speculative model of Amt function in olfactory sensilla. (A) Diagram of an ac1 sensillum in a control fly. Prior to ammonia stimulation (left panel), extremely low ambient levels of ammonia (blue stars) are maintained in the sensillum lymph due to the activity of Amt proteins (green) in auxiliary cells. As a result, neither ammonia receptors (red) nor their associated ORN (orange) is activated. Like most ORNs, the neuron exhibits some spontaneous activity. During ammonia stimulation (right panel), ammonia enters the sensillum through pores in the cuticle and binds ammonia receptors to activate a barrage of action potentials in the ORN. (B) Diagram of an ac1 sensillum in an *Amt^1^* mutant. Due to the lack of Amt in auxiliary cells, the concentration of ammonia in the sensillar lymph is higher than in control flies (left). These ammonia levels are sufficient to desensitize the ammonia receptors and/or the ammonia-sensitive neuron (faded orange), which in turn lowers the spontaneous activity. During ammonia stimulation (right), the desensitized state of the receptor and/or neuron prevents neuronal activation.
(TIFF)

Table S1 Summary of *Drosophila* antennal RNA-Seq datasets. Total reads are the number of reads passing quality control for each sample. Aligned reads and Percent aligned are the number and percent of total reads that could be aligned to the *Drosophila* reference genome (BDGP Release 5) or a splice junction set (see Materials and Methods). Of these aligned reads, the majority were mapped to genomic regions associated with FlyBase genes (Reads mapped to genes and Percent mapped reads).
(PDF)

Dataset S1 Antennal expression and differential gene expression in CS and *ato* flies. This dataset contains four spreadsheets summarizing the raw data from our third antennal segment dataset. 1) The spreadsheet “by exon” lists each mapped exon of the 14,078 mapped genes. The exons are listed by chromosome, exon start and stop locations, and associated gene by its gene symbol at the time of the analysis. The raw number of reads in each sample is provided for the three *ato* and three CS samples. Chromosomal regions that are annotated with more than one gene were considered ambiguous and were not analyzed. *2)* The spreadsheet “by splice” reports the number of reads mapping to splice junctions between particular exons in each sample, listing the chromosome, the start site of the intron, the end site of the intron, a name for the splice junction that includes the gene symbol, and the number of reads mapped across that splice junction for that sample. For each sample, only splice junctions to which reads were mapped are listed. *3)* The spreadsheet “by gene”, lists each of the 14,078 mapped genes by their symbol, then the total number of raw reads mapped to that gene, ie the sum of the reads mapped to exons and splice junctions, for each of the three *ato* and three CS samples. This section is highlighted in blue. In the orange section, the reads per million mapped reads (RPM) is reported for each lane. The RPM normalizes the total number of genes detected in each lane. This value needed to be >1 for a gene to be considered detected in that sample. In the green section, gene symbol, bp of gene length, and the RPM per kilobase of gene length (RPKM) is reported for each sample. After a vertical dividing line, the average RPKM for each gene is reported for each genotype, along with the SEM. Finally, the current gene symbols and FBgn numbers are provided in the purple column. *4)* The spreadsheet “differential expression” summarizes the EdgeR differential expression analysis. In the orange section, each of the 9,034 antennal expressed genes is listed, followed by the log of the ratio of average expression in CS and *ato* samples (log_2_(RPM*_ato-_*_avg_ */*RPM_CS-avg_)). The next columns list each gene's average expression in all six samples on a log basis (log_2_(RPM)), its p-value for differential expression between the two genotypes, its false discovery rate (FDR), its current gene symbol and current FBgn number. Note that the RPM values are based on the EdgeR analysis which adds a small value to each gene's RPM value to avoid calculating infinite log fold changes for samples with zero reads. See Materials and Methods for further details. Additionally, the EdgeR analysis normalizes the libraries to account for gene distribution (see EdgeR documentation for further details). For these reasons, the EdgeR log_2_(RPM) reported on this spreadsheet is close to, but not exactly the same as, the one that would be calculated from the raw RPM values in the “by gene” spreadsheet. The blue section reports the 250 genes whose expression was significantly reduced at least 4-fold in *ato* flies. These were considered the *ato*-depleted genes. In the green section, the 155 genes that were significantly enriched at least 4-fold in *ato* flies compared to CS flies are reported.
(XLSB)
