# Supplementary figures and images for: An RNA-Seq Screen of the Drosophila Antenna Identifies a Transporter Necessary for Ammonia Detection
Source: PLoS Genet. 2014 Nov 20;10(11):e1004810. doi: 10.1371/journal.pgen.1004810 (PMC4238959; doi:10.1371/journal.pgen.1004810)

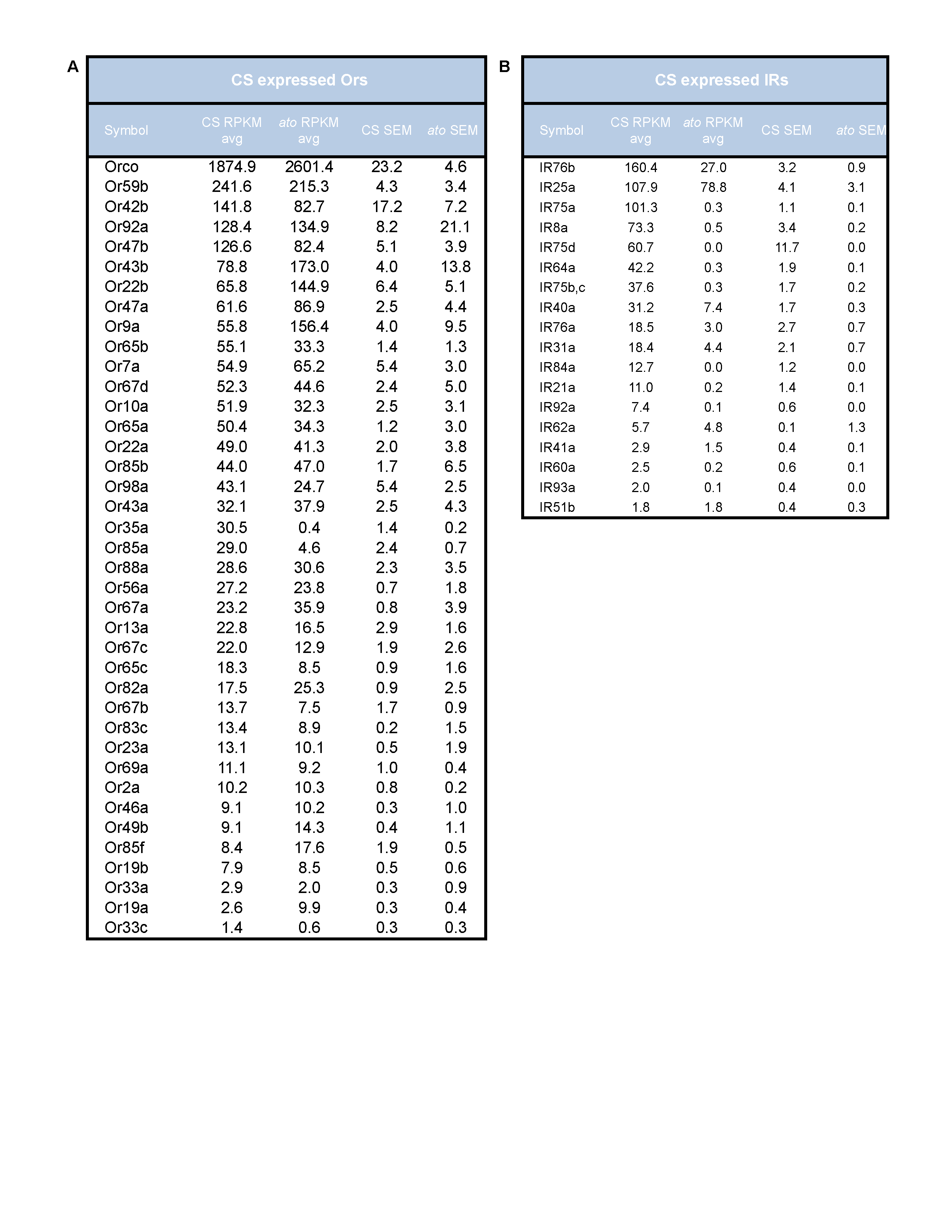

Supplement: Figure S1 — Olfactory receptor gene expression in CS and ato flies. (A) The 39 Ors and (B) 19 IRs expressed in CS fly antennae are listed by descending expression level (RPKM), averaged across the three samples. Like other genes, the olfactory receptor genes are considered expressed in CS if there were at least 1 RPM in each of the three CS samples. For comparison, the average expression levels of the same olfactory receptors are provided for ato flies. (TIFF) [file pgen.1004810.s001.tiff]

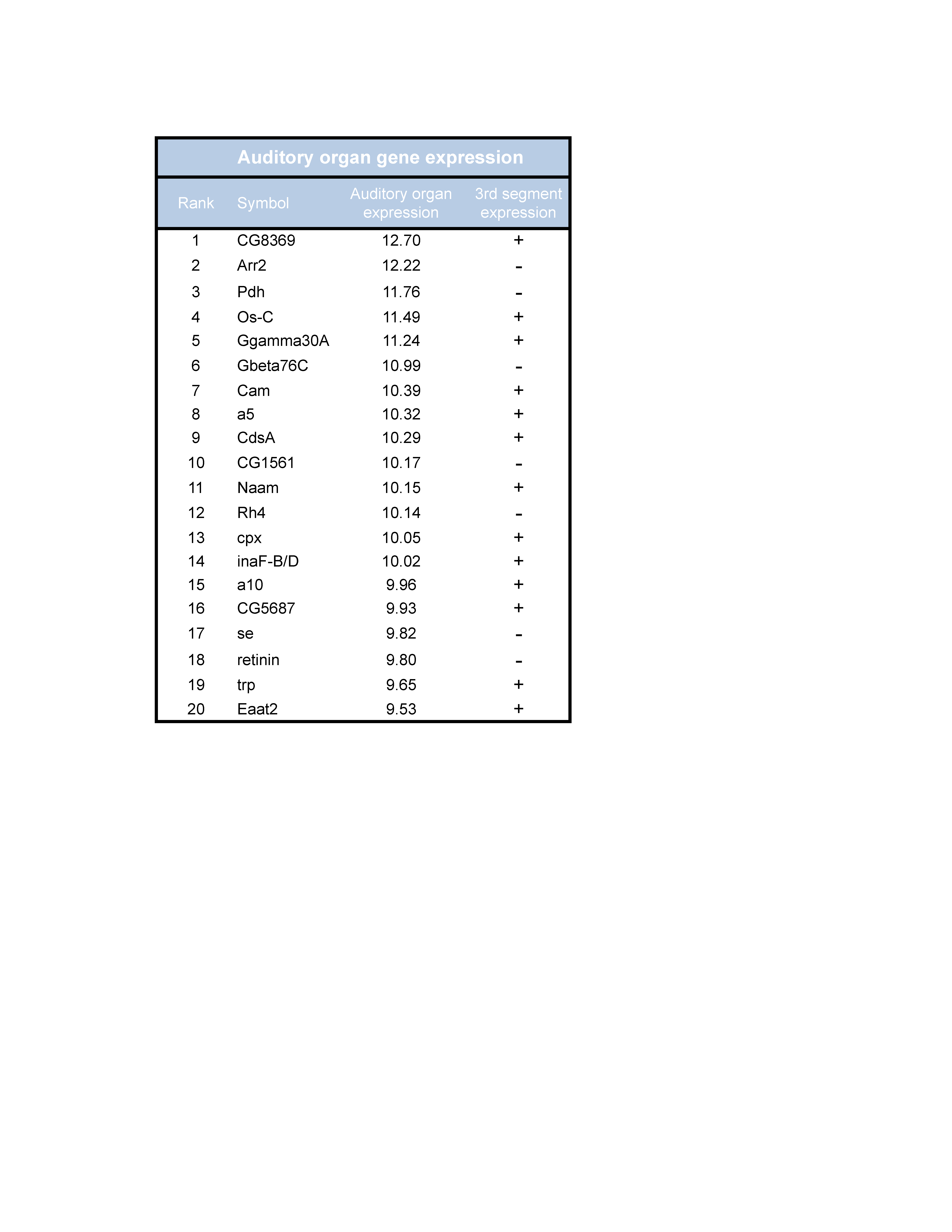

Supplement: Figure S2 — Auditory organ gene expression in the third antennal segment. The 20 most highly expressed auditory organ genes are listed in descending order by their expression level (data from[27]). The expression of auditory organ genes was determined by averaging the mean microarray fluorescence intensities from the six “control” replicates of each of the 274 auditory organ genes listed in Table S2 of Senthilan et al. 2012. “+” indicates genes that were detected at >1 RPM in each of our three CS third antennal segment samples, i.e. genes that met our standard criteria for expression in CS. Seven of the 20 genes were not considered expressed, suggesting that the auditory organ of the antenna, the second antennal segment, did not substantially contaminate our collection of the olfactory third antennal segments. (TIFF) [file pgen.1004810.s002.tiff]

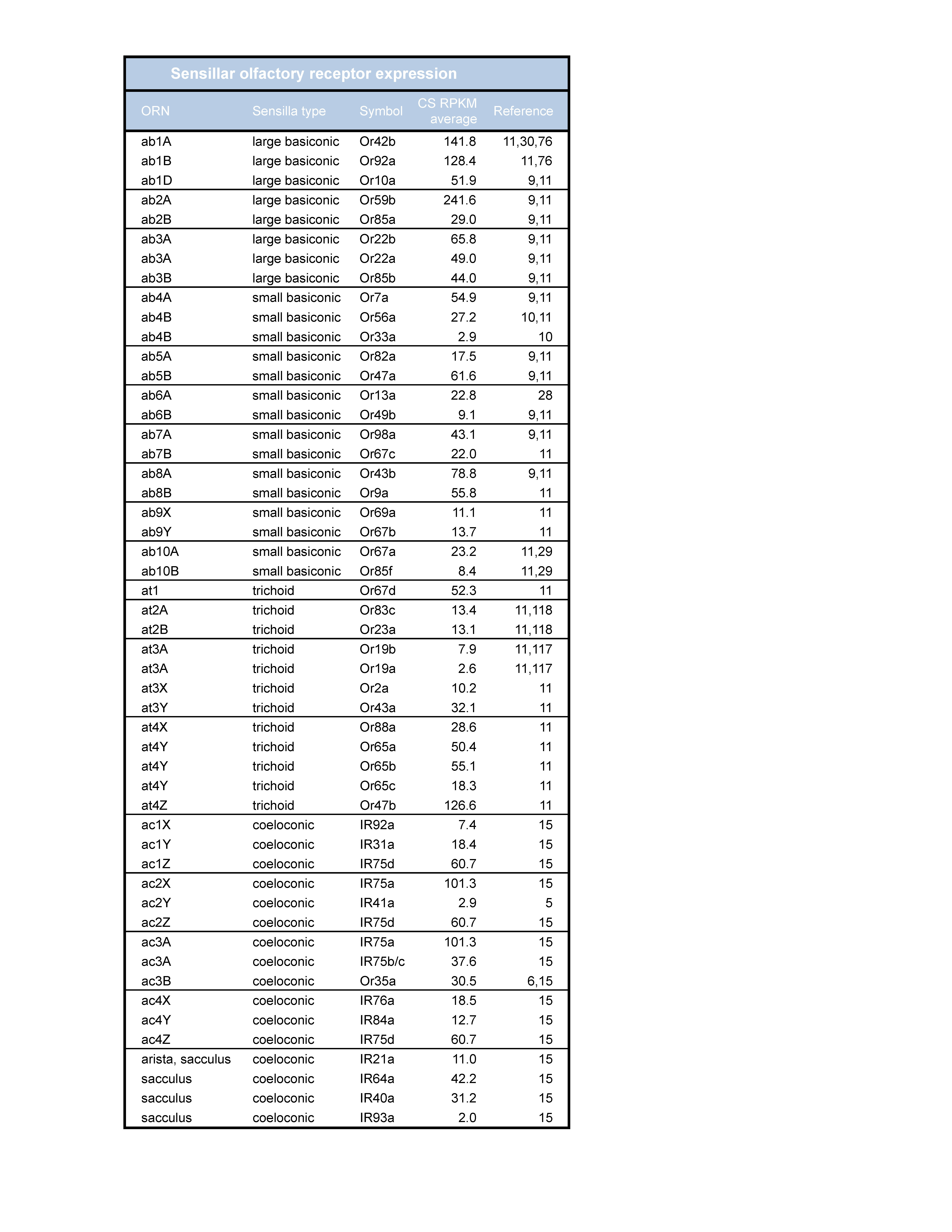

Supplement: Figure S3 — Olfactory receptor expression by sensillum type and ORN class. In Drosophila, olfactory sensilla house 1–4 ORNs that can be distinguished by their odor response profiles. Stereotyped groupings of ORNs form different sensilla types. Individual ORNs are designated by their sensillar morphology and type: large basiconic (ab1–3), small basiconic (ab4–10), trichoid (at1–4), or coeloconic (ac1–4) sensilla. Additionally, many ORNs are designated by the relative size of their spike amplitude, such that the A neuron has the largest amplitude spikes within a sensillum type, the B neuron has the next largest, etc. Previous studies have mapped individual olfactory receptors to specific sensilla types, and in many cases to particular ORNs [5], [6], [9]–[11], [15], [28]–[30], [76], [117], [118]. This table lists the average expression level of each olfactory receptor detected in CS flies by its sensillar type and ORN. In some sensillum types, specific ORNs have not been identified, and we use the suffixes X, Y, and Z to designate the different ORNs in order to clarify which receptors are co-expressed in individual ORNs. Different sensillum types are separated by a thin line. Some IRs are found in multiple coeloconic types and are listed more than once, and IRs found in the sacculus and artista are also listed. The broadly expressed co-receptors Orco, IR8a, IR25a and IR76b are not listed here. (TIFF) [file pgen.1004810.s003.tiff]

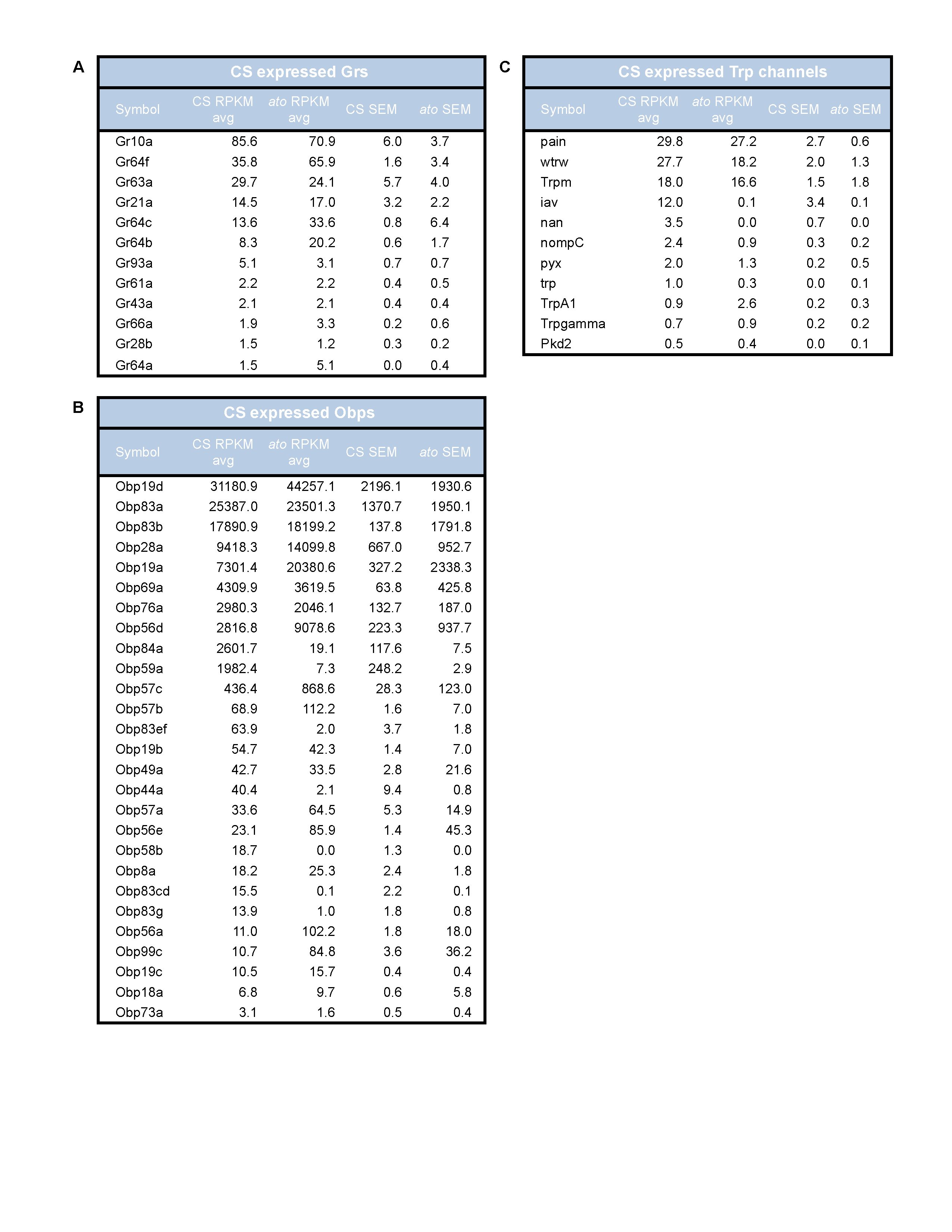

Supplement: Figure S4 — Chemosensory gene expression in CS and ato flies. (A) 12 Gr genes are expressed in CS antennae and are listed by descending expression level (RPKM) averaged across samples. Their expression levels in ato are also indicated. (B) CS-expressed Obps are detected at a wide range of expression, and a subset are substantially reduced in ato flies. We note that Obp76a is formally known as lush. (C) Most Drosophila Trp channel family members are detected in CS antennae, albeit at relatively low levels, and most are found at similar levels in ato flies. (TIFF) [file pgen.1004810.s004.tiff]

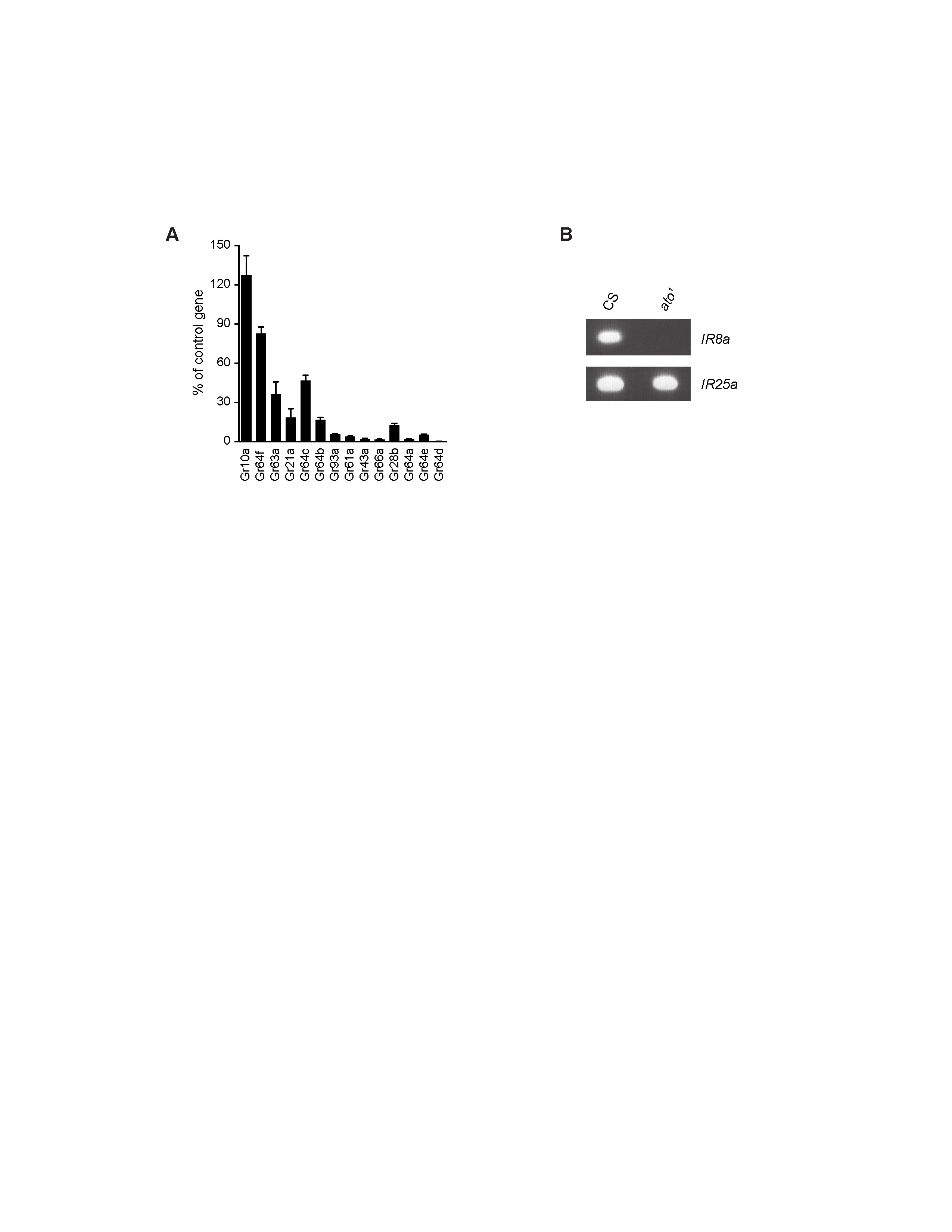

Supplement: Figure S5 — Validation of chemosensory gene expression. (A) Expression of 14 Gr genes was verified by qRT-PCR using antennal cDNA. Expression was quantified relative to a control gene amplified in all reactions, and the Gr genes are listed in the same order as in Figure S1F to facilitate comparison of relative levels of gene expression. Gr64d and Gr64e are also detected in the CS antenna by qRT-PCR and are listed last. (B) RT-PCR on CS antennal cDNA indicates that IR8a expression is lost in ato flies, whereas substantial expression of IR25a remains. Both genes are found in CS antennae. (TIFF) [file pgen.1004810.s005.tiff]

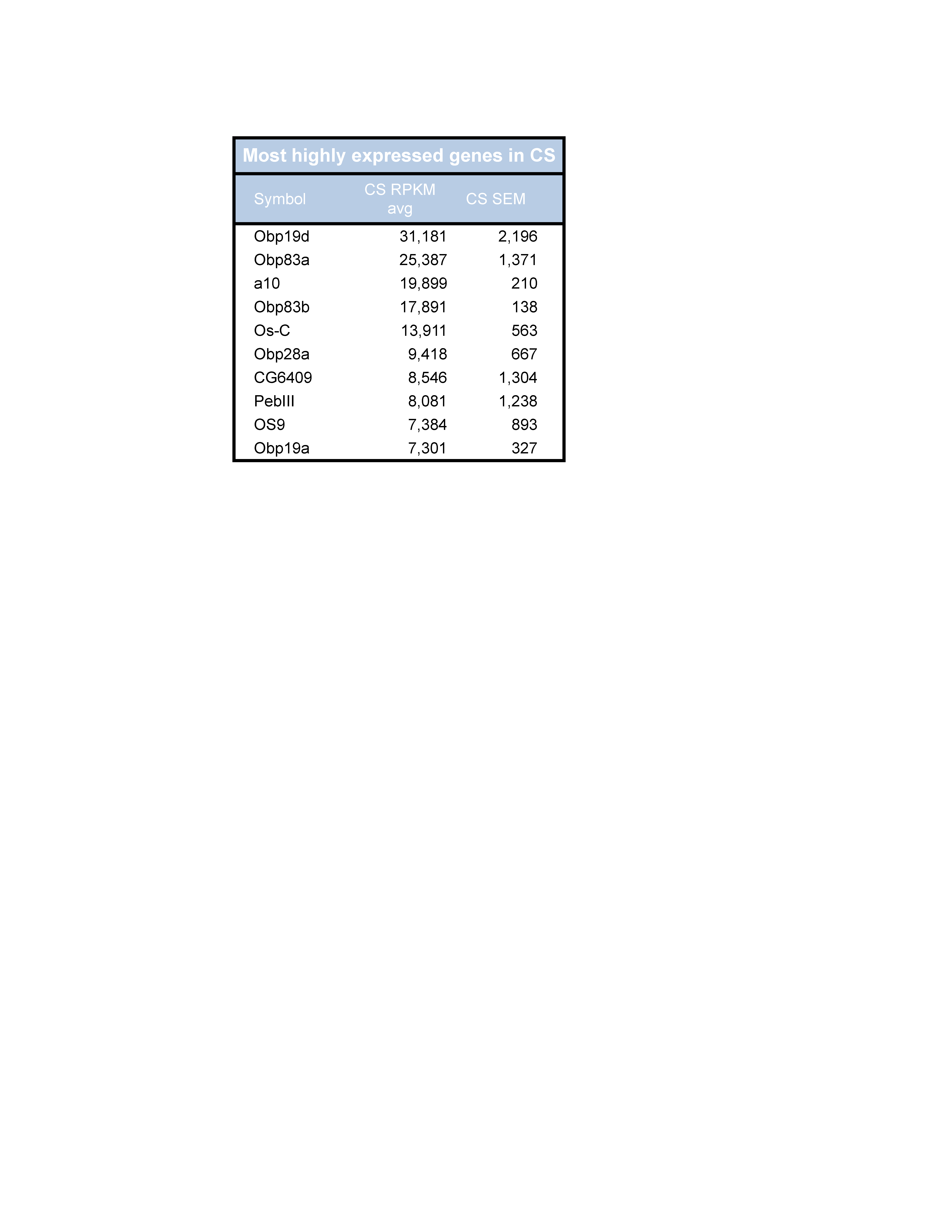

Supplement: Figure S6 — Genes most highly expressed in CS antennae. The ten genes with the highest expression levels in CS antennae are listed in decreasing order of expression level (by RPKM). (TIFF) [file pgen.1004810.s006.tiff]

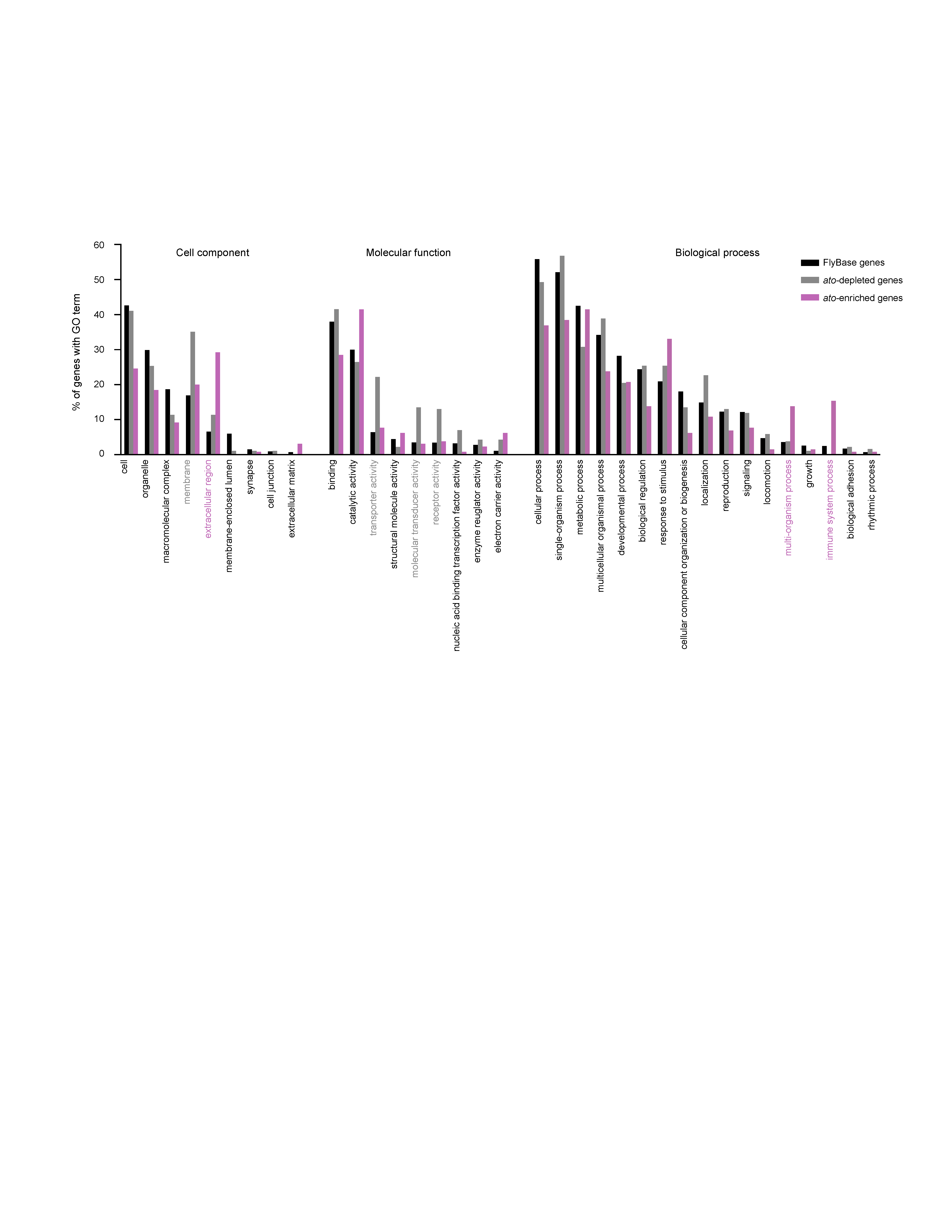

Supplement: Figure S7 — Gene ontology terms enriched in CS and ato fly antennae. Gene ontology (GO) terms represent gene product properties in three categories: Cell component, Molecular function, and Biological process. These three descriptors are called “level 1” terms. Lower level terms are more specific and form a subset of the higher level terms. These subsets overlap, and thus a gene can be annotated with multiple different GO terms, such as “binding” and “receptor activity” within Molecular function. Many, but not all, individual genes have been either manually or automatically annotated with GO terms. In total, 13,737 Flybase D. melanogaster genes, 185 of 250 ato-depleted genes, and 130 of 155 ato-enriched genes have been annotated with at least one GO term. The graph depicts the percentage of the annotated genes in each group that are annotated with each level 2 GO term. In general, the percentage of genes annotated with each GO term was comparable between groups. However, the GO analysis program AmiGO detected a significantly higher proportion of the 185 ato-depleted genes associated with the GO terms “membrane”, “transporter activity”, “molecular transducer activity”, and “receptor activity” compared to the FlyBase genes (see also Figure 3). Significant enrichment of the terms “extracellular region”, multi-organism process”, and “immune system process” were detected in the 130 ato-enriched genes compared to the FlyBase genes. For simplicity, level 2 GO terms were only included if at least 1% of genes in one of the three groups are annotated with a given GO term. (TIFF) [file pgen.1004810.s007.tiff]

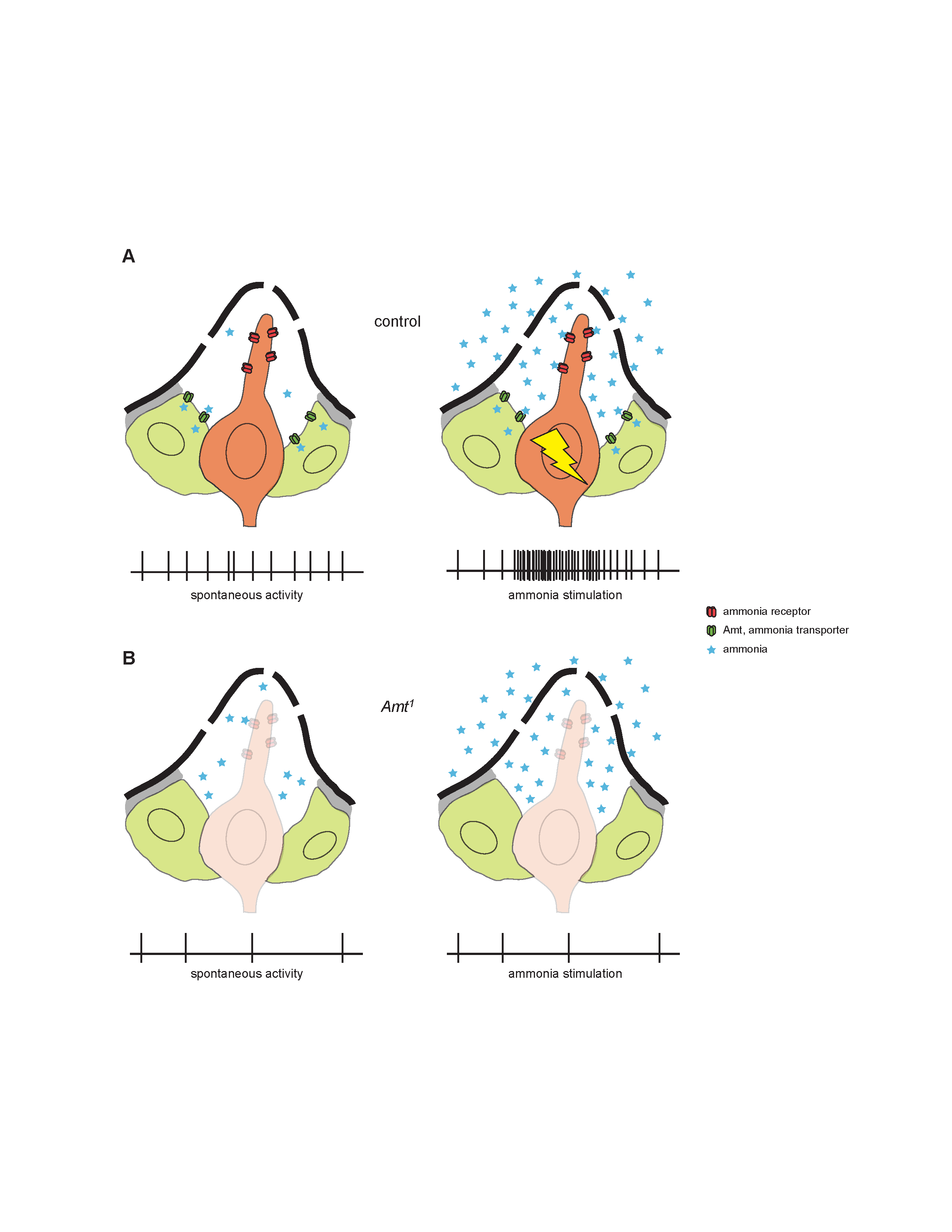

Supplement: Figure S8 — A speculative model of Amt function in olfactory sensilla. (A) Diagram of an ac1 sensillum in a control fly. Prior to ammonia stimulation (left panel), extremely low ambient levels of ammonia (blue stars) are maintained in the sensillum lymph due to the activity of Amt proteins (green) in auxiliary cells. As a result, neither ammonia receptors (red) nor their associated ORN (orange) is activated. Like most ORNs, the neuron exhibits some spontaneous activity. During ammonia stimulation (right panel), ammonia enters the sensillum through pores in the cuticle and binds ammonia receptors to activate a barrage of action potentials in the ORN. (B) Diagram of an ac1 sensillum in an Amt1 mutant. Due to the lack of Amt in auxiliary cells, the concentration of ammonia in the sensillar lymph is higher than in control flies (left). These ammonia levels are sufficient to desensitize the ammonia receptors and/or the ammonia-sensitive neuron (faded orange), which in turn lowers the spontaneous activity. During ammonia stimulation (right), the desensitized state of the receptor and/or neuron prevents neuronal activation. (TIFF) [file pgen.1004810.s008.tiff]
