# Supplementary material for: An RNA-Seq Screen of the Drosophila Antenna Identifies a Transporter Necessary for Ammonia Detection
Source: PLoS Genet. 2014 Nov 20;10(11):e1004810. doi: 10.1371/journal.pgen.1004810 (PMC4238959; doi:10.1371/journal.pgen.1004810)
Supplement: Table S1 — Summary of Drosophila antennal RNA-Seq datasets. Total reads are the number of reads passing quality control for each sample. Aligned reads and Percent aligned are the number and percent of total reads that could be aligned to the Drosophila reference genome (BDGP Release 5) or a splice junction set (see Materials and Methods). Of these aligned reads, the majority were mapped to genomic regions associated with FlyBase genes (Reads mapped to genes and Percent mapped reads). (PDF) [file pgen.1004810.s009.pdf]

Table S1: Summary of *Drosophila* antennal RNA-Seq datasets

Sample	Total reads	Aligned reads	Percent aligned	Reads mapped to genes	Percent mapped reads
CS 1	7,871,831	6,747,381	85.7%	6,043,268	89.6%
CS 2	8,439,162	7,073,720	83.8%	6,391,294	90.4%
CS 3	7,911,591	6,796,317	85.9%	6,057,179	89.1%
ato 1	4,657,572	4,272,840	91.7%	4,024,370	94.2%
ato 2	6,524,293	5,982,528	91.7%	5,573,418	93.2%
ato 3	8,271,873	7,642,501	92.4%	7,126,208	93.2%
